# Band Degeneracy and Anisotropy Enhances Thermoelectric Performance from Sb_2_Si_2_Te_6_ to Sc_2_Si_2_Te_6_

**DOI:** 10.1021/jacs.4c01838

**Published:** 2024-06-18

**Authors:** Wenzhen Dou, Kieran B. Spooner, Seán R. Kavanagh, Miao Zhou, David O. Scanlon

**Affiliations:** † School of Physics, Beihang University, Beijing 100191, China; ‡ Department of Chemistry, University College London, London WC1H 0AJ, U.K.; § School of Chemistry, University of Birmingham, Birmingham B15 2TT, U.K.; ∥ John A. Paulson School of Engineering and Applied Sciences, Harvard University, Cambridge, Massachusetts 02138, United States; ⊥ Thomas Young Centre, University College London, London WC1E 6BT, U.K.; # Hangzhou International Innovation Institute, Beihang University, Hangzhou 311115, China; ¶ Tianmushan Laboratory, Hangzhou 310023, China

## Abstract

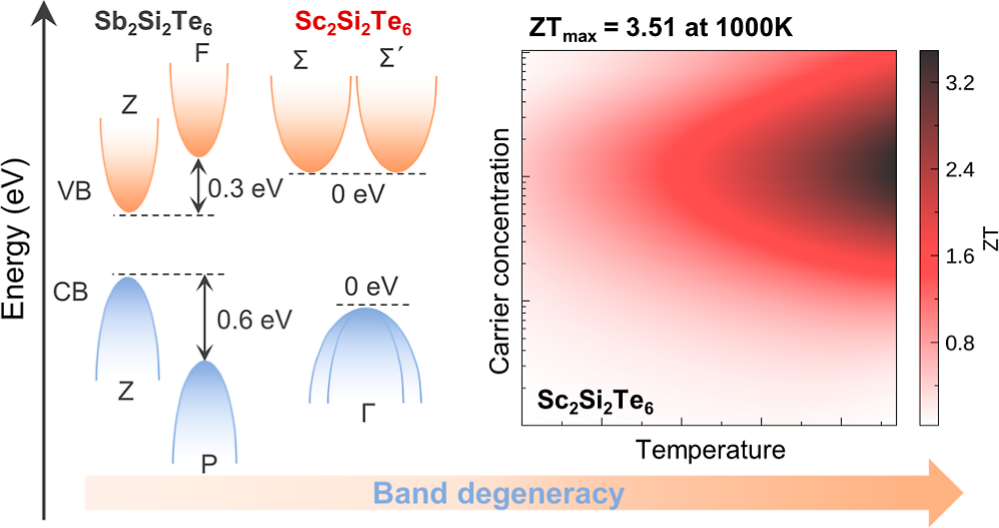

The complex interrelationships among thermoelectric parameters mean that *a priori* design of high-performing materials is difficult. However, band engineering can allow the power factor to be optimized through enhancement of the Seebeck coefficient. Herein, using layered Sb_2_Si_2_Te_6_ and Sc_2_Si_2_Te_6_ as model systems, we comprehensively investigate and compare their thermoelectric properties by employing density functional theory combined with semiclassical Boltzmann transport theory. Our simulations reveal that Sb_2_Si_2_Te_6_ exhibits superior electrical conductivity compared to Sc_2_Si_2_Te_6_ due to lower scattering rates and more pronounced band dispersion. Remarkably, despite Sb_2_Si_2_Te_6_ exhibiting a lower lattice thermal conductivity and superior electrical conductivity, Sc_2_Si_2_Te_6_ is predicted to achieve an extraordinary dimensionless figure of merit (*ZT*) of 3.51 at 1000 K, which significantly surpasses the predicted maximum *ZT* of 2.76 for Sb_2_Si_2_Te_6_ at 900 K. We find the origin of this behavior to be a combined increase in band (valley) degeneracy and anisotropy upon switching the conduction band orbital character from Sb p to Sc d, yielding a significantly improved Seebeck coefficient. This work suggests that enhancing band degeneracy and anisotropy (complexity) through compositional variation is an effective strategy for improving the thermoelectric performance of layered materials.

## Introduction

With the development of electronics and ever-increasing demand for sustainable and versatile energy harvesting, thermoelectrics have become the subject of rapidly growing interest because they can directly transform thermal energy into valuable electrical power.^1^ The efficiency of thermoelectric materials is quantified by the dimensionless figure of merit *ZT*, defined as *ZT* = *S*^2^σ*T*/κ, where *S* is the Seebeck coefficient, σ represents the electrical conductivity, and *T* denotes the absolute temperature. The thermal conductivity κ includes both lattice thermal conductivity κ_l_ and the electronic thermal conductivity κ_e_. An ideal thermoelectric material should simultaneously exhibit high electrical conductivity to minimize internal energy loss, a high Seebeck coefficient for generating high voltage, and low thermal conductivity to maintain the temperature gradient. Motivated by these design principles, considerable efforts in the past decade have been devoted to exploring potential candidates that inherently possess the crucial attributes required for thermoelectric applications.^2,3^ Among the rapidly growing class of thermoelectric materials, layered compounds have shown advantages due to their unique crystal structure characterized by strong intralayer and weak interlayer bonds.^4−8^ First, their strong discrepancy in chemical bonding strength is believed to induce partially localized low-frequency phonon modes, low phonon group velocities, and large anharmonicity—all favorable for achieving low lattice thermal conductivity.^9−11^ Moreover, the low-dimensional structure translates to reduced dimensionality in the electrical transport properties, which can benefit thermoelectric performance.^12,13^ Furthermore, the anisotropic behavior of electron and phonon transport in these layered systems provides an excellent opportunity to disentangle the interconnected thermoelectric parameters, especially when thermoelectric conversion occurs along the desired crystal direction within a single crystal.^14,15^

Within the layered compounds, the A_2_B_2_Q_6_ family (A = In, Cr, Bi, Sb, or Sc; B = Si, Ge; and Q = Se, Te) has been intensively explored,^16−24^ and some compounds show intriguing magnetic and magnetoelectric properties, such as pressure-driven spin-crossover and long-range ferromagnetic order.^19,20^ Interestingly, compounds based on A = Cr, Sb, In, and Bi are also reported to be promising candidates for thermoelectric applications.^4,17,25,26^ In particular, Sb_2_Si_2_Te_6_ has been the focus of interest due to its remarkable thermoelectric properties.^4,27^ Sb_2_Si_2_Te_6_ adopts a layered two-dimensional structure composed of Sb^3+^ cations and [Si_2_Te_6_]^6–^ units, resembling the Fe_2_P_2_Se_6_ structure type. Owing to a low phonon group velocity and strong anharmonicity, Sb_2_Si_2_Te_6_ exhibits a relatively low thermal conductivity, approximately 1.3 W m^–1^ K^–1^ at room temperature, decreasing to 0.5 W m^–1^ K^–1^ at 823 K.^4^ Consequently, Sb_2_Si_2_Te_6_ achieves an impressive *ZT* of 1.08 at 823 K.^4^ Additionally, by constructing a cellular nanostructure through the deposition of a thin layer of Si_2_Te_3_ onto Sb_2_Si_2_Te_6_ grains, the peak *ZT* at 823 K can be improved to 1.65 in the Sb_2_Si_2_Te_6_/Si_2_Te_3_ cellular network.^4^ Nevertheless, the average power factor (PF) of Sb_2_Si_2_Te_6_—approximately 10 μW cm^–1^ K^–2^ within the temperature range of 400–823 K—lags behind that of conventional Pb-based thermoelectric materials (∼30 μW cm^–1^ K^–2^ for p-type PbTe and ∼20 μW cm^–1^ K^–2^ for n-type PbTe).^28−30^ As a result, it is highly desirable to enhance the PF and, by extension, the overall thermoelectric performance of Sb_2_Si_2_Te_6_-based thermoelectric devices.

Band engineering strategies are widely employed to improve the thermoelectric performance of materials.^31−34^ For the most part, the relationship between electronic band structure and thermoelectric performance (assuming a given doping concentration) can be approximately understood through the density of states carrier effective mass  and the conductivity effective mass —corresponding to the geometric and harmonic means of the carrier effective masses in each direction *i* (with *N* dimensions). A larger density of states effective mass yields a larger Seebeck coefficient (*S*), while a smaller conductivity effective mass benefits electrical conductivity (σ), both of which contribute to improved thermoelectric performance as seen through the *ZT* figure of merit definition
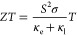


Two key mechanisms through which band structure engineering can thus boost thermoelectric performance, via effects on *m*_DOS_^*^ and *m*_σ_^*^, are through changes in band anisotropy and band degeneracy. Anisotropy in the electronic band structure (i.e., a combination of light and heavy carrier masses for different propagation directions) results in a disparity in *m*_DOS_^*^ and *m*_σ_^*^, which otherwise are equal for isotropic bands. This allows the combination of a larger density of states mass *m*_DOS_^*^ with a smaller conductivity mass *m*_σ_^*^, which can boost *ZT* by up to a factor of 3 as compared to isotropic bands.^35^

Band degeneracy, in particular, can enhance the Seebeck coefficient, and thus the ultimate thermoelectric performance, by increasing the density of states mass *m*_DOS_^*^.^36,37^ Band degeneracy refers to the case where multiple bands have identical (degenerate) or closely matching within a few *k*_B_T (effectively degenerate) energies. This results in an increased density of states effective mass according to ,^38^ where *N*_V_ is the number of degenerate band edges and m* is the corresponding single-band effective mass. Band degeneracy can manifest in semiconductors via two primary mechanisms. The first, termed orbital degeneracy, occurs when multiple electronic bands have minimal (or no) energy difference at the same band extrema positions in reciprocal space (such as the triply degenerate *t_2g_* levels for *d*-orbitals in an octahedral crystal field). The second, termed valley or *k*-point degeneracy, occurs when multiple valleys (band extrema at different *k*-points) of the same electronic bands in the Brillouin zone are (effectively) degenerate, often due to symmetry equivalence via the combination of crystal and orbital symmetries (such as the double-well inverted band structure of Bi_2_Te_3_).^39^ Numerous studies have demonstrated that band degeneracy can be enhanced by tuning the material composition, as exemplified in the Mg_2_Si_1–*x*_Sn_*x*_^36^ and Bi_2–*x*_Sb_*x*_Se_3_ solid solutions.^40^ Recently, Wang et al. reported a 22% improvement in the *ZT* due to enhanced band degeneracy upon substituting p-valent Bi in BiCuOSe with d-valent La, predicting a *ZT* value of 1.46 for n-type LaCuOSe.^41^ Inspired by this observation of enhanced band degeneracy upon heterovalent substitution, we investigate the effect on thermoelectric performance in the A_2_B_2_Q_6_ family by replacing p-valent Sb with a d-valent cation. Several members of the A_2_B_2_Q_6_ family have been experimentally reported thus far, including Sc_2_Si_2_Te_6_ in 2022^16^—however, the thermoelectric properties were not investigated. We note that the thermoelectric properties of Sc_2_Si_2_Te_6_ monolayers were recently theoretically investigated, for which high *ZT* values were predicted.^42^

In this work, we investigate the effects on band structure and thermoelectric performance by modifying the conduction band orbital character in this crystal family, shifting from a Sb *p* derived CBM in Sb_2_Si_2_Te_6_ to a Sc *d* CBM in Sc_2_Si_2_Te_6_. Sc_2_Si_2_Te_6_ shares an identical stacking fault pattern with Sb_2_Si_2_Te_6_ and crystallizes in the same rhombohedral symmetry (space group *R*3̅) with a single distinct crystallographic position each for Sc, Si, and Te as revealed experimentally by high-resolution transmission electron microscopy and diffraction patterns.^16^ We systematically investigated the thermal and electrical transport properties in layered Sb_2_Si_2_Te_6_ and Sc_2_Si_2_Te_6_ by solving the Boltzmann transport equation based on first-principles calculations, revealing a significant enhancement of band degeneracy and, consequently, the Seebeck coefficient in Sc_2_Si_2_Te_6_ compared to Sb_2_Si_2_Te_6_. We predict n-type Sc_2_Si_2_Te_6_ to achieve an optimal *ZT* of 3.51 at 1000 K, surpassing the maximum *ZT* of 2.76 obtained for p-type Sb_2_Si_2_Te_6_ by 27%. These findings demonstrate that Sc_2_Si_2_Te_6_ presents a competitive alternative to Sb_2_Si_2_Te_6_, highlighting the potential of band structure engineering in optimizing the PF and thermoelectric performance.

## Results and Discussion

### Equilibrium Geometry and Electronic Structure

**Figure 1:**
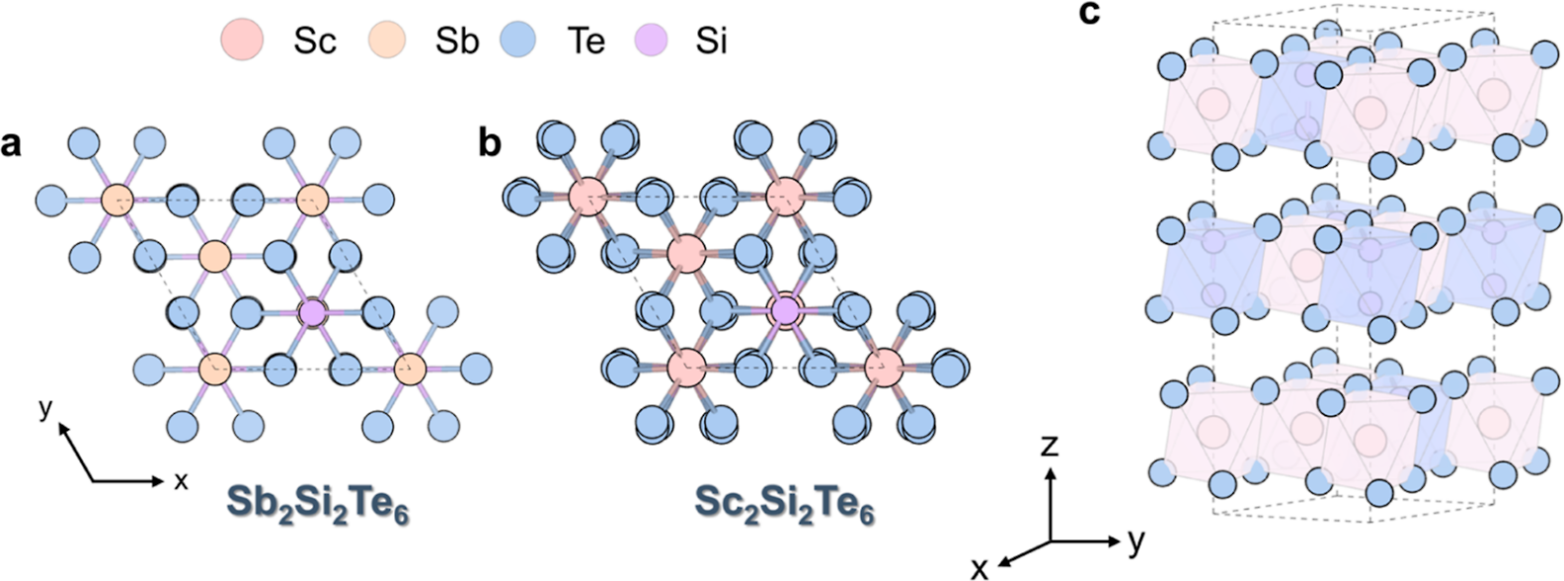
Top view of the crystal structure for (a) Sb_2_Si_2_Te_6_ and (b) Sc_2_Si_2_Te_6_. The conventional unit cell is indicated by gray dashed lines. (c) Side view of Sc_2_Si_2_Te_6_. There is a slight asymmetric distortion of the octahedra in Sc_2_Si_2_Te_6_. The atom colors are Sb: orange, Sc: pink, Si: purple, and Te: blue.

Both Sb_2_Si_2_Te_6_ (Figure 1a) and Sc_2_Si_2_Te_6_ (Figure 1b) exhibit rhombohedral symmetry (space group *R*3̅, no. 148) and consist of ABC-stacked slabs of (Sb/Sc)_2_Si_2_Te_6_, where Sb (Sc) atoms and Si–Si dumbbells are located in Te-coordinated octahedra within the slabs (Figure 1c)—which individually have hexagonal symmetry. Table 1 presents the lattice parameters of the conventional unit cell, optimized using both PBEsol and HSE06 with the D3 dispersion correction. The calculated lattice constants for Sb_2_Si_2_Te_6_ and Sc_2_Si_2_Te_6_ agree well with the experimental measurements,^4,16^ with HSE06 + D3 showing improved agreement, though with a slight underestimation of the interlayer spacing as expected when neglecting temperature effects.^43,44^ Compared to Sb_2_Si_2_Te_6_, Sc_2_Si_2_Te_6_ exhibits a slight asymmetric distortion of the octahedra, with shorter Sc–Te bond lengths due to the smaller ionic radius of Sc but larger interlayer spacing.

**Table 1:** Calculated Crystal Structure Information of Sb_2_Si_2_Te_6_ and Sc_2_Si_2_Te_6_^a^

functional	compound	*a* (Å)	*c* (Å)	Sb/Sc–Te (Å)	*d*_layer_ (Å)
PBEsol + D3	Sb_2_Si_2_Te_6_	7.07 (−1.4%)	20.10 (−5%)	3.05	2.78
	Sc_2_Si_2_Te_6_	6.85 (−2%)	20.16 (−5%)	2.90	2.99
HSE06 + D3	Sb_2_Si_2_Te_6_	7.07 (−1.4%)	20.67 (−2.4%)	3.04	3.01
	Sc_2_Si_2_Te_6_	6.91 (−1.2%)	20.81 (−2%)	2.93	3.22

a The % differences to the experimental structures are given in parentheses.^4,16^*d*_layer_ is the interlayer distance.

Using HSE06 + SOC with the doped package,^45^ the stability region (chemical potential limits) of Sc_2_Si_2_Te_6_ in the Sc–Si–Te chemical space was computed (Figure S1), showing it to be thermodynamically stable with a relatively large stability window, suggesting ready synthesizability as witnessed experimentally.^16^

**Figure 2:**
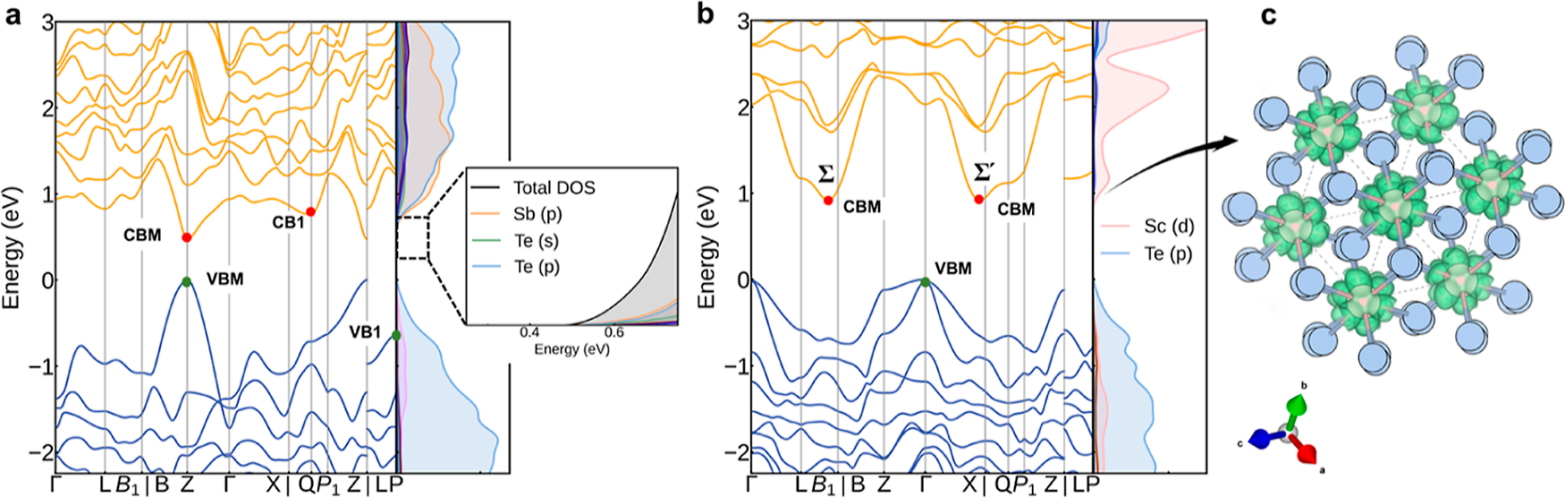
Calculated electronic band structures and orbital-projected DOS of (a) Sb_2_Si_2_Te_6_ and (b) Sc_2_Si_2_Te_6_ using HSE06 + SOC along the high-symmetry *k*-point path for rhombohedral crystals within the Setyawan and Curtarolo convention.^46^ The VBM is set to zero. (c) Charge density isosurface of the Sc_2_Si_2_Te_6_ CBM state. The isosurface level is set to 0.02 e/Å^3^.

Figure 2 displays the calculated electronic band structures and DOS of Sb_2_Si_2_Te_6_ and Sc_2_Si_2_Te_6_ using HSE06 + SOC. The difference in A-site cations markedly affects the band structure. Sb_2_Si_2_Te_6_ exhibits a direct band gap of 0.44 eV, with both the CBM and VBM located at the *Z* point, and this value is close to the experimental optical gap (∼0.6 eV).^4^ In contrast, Sc_2_Si_2_Te_6_ exhibits an indirect band gap of 0.92 eV, where the VBM is located at the Γ point and the CBM is at the Σ point (1/2, −1/6, 1/6) along the L–B path in reciprocal space. This is not a high-symmetry *k*-point, resulting in significantly increased *k*-point (valley) degeneracy at the conduction band edge, as discussed below.^32^ Sb_2_Si_2_Te_6_ has high electronic band dispersion, which will yield small carrier effective masses and higher carrier mobility. As indicated by the accompanying DOS plots in Figure 2, the CBM is composed of Sb 5*p* and Te 5*p* orbitals in Sb_2_Si_2_Te_6_ and Sc 3*d* orbitals in Sc_2_Si_2_Te_6_, while the VBM mainly derives from Te 5*p* orbitals. In Sb_2_Si_2_Te_6_, there is a slight contribution to the VBM from Sb 5*s* orbitals, while there is minimal hybridization with Sc 3*d* orbitals at the VBM in Sc_2_Si_2_Te_6_, which modifies the Te *p* valence band edge. Consequently, the VBM in Sc_2_Si_2_Te_6_, located at Γ (rather than *Z*), is doubly (rather than singly) degenerate and exhibits reduced band dispersion. As expected, due to the presence of heavy elements Te and (to a lesser extent) Sb, spin–orbit coupling (SOC) is found to significantly reduce the band gaps of these compounds, contributing VBM upshifts of 0.2–0.3 eV (Figure S2).

**Figure 3:**
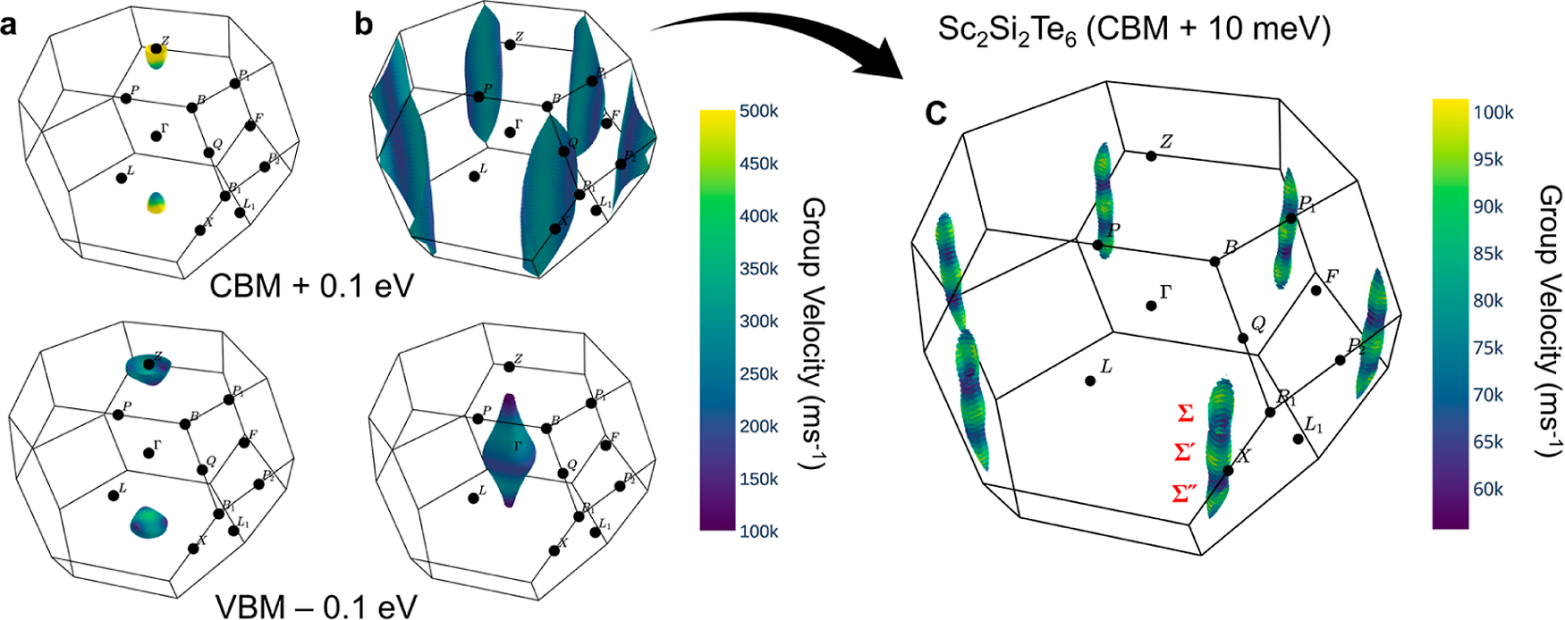
Fermi surface of (a) Sb_2_Si_2_Te_6_ and (b) Sc_2_Si_2_Te_6_ plotted at 0.1 eV below VBM and above CBM, respectively. (c) Iso-energy Fermi surface is 10 meV above the CBM for Sc_2_Si_2_Te_6_. The different colors represent the magnitude of carrier group velocities.

It is noteworthy that Sb_2_Si_2_Te_6_ exhibits an energy difference of 0.3 eV between the lowest two conduction band valleys (i.e., *E*_CB1_ – *E*_CBM_) and 0.6 eV between the highest two valence band valleys (i.e., *E*_VBM_ – *E*_VB1_). In contrast, Sc_2_Si_2_Te_6_ features two degenerate CBM minima [Σ and Σ′ at (1/2, −1/6, 1/6) and (1/3, −1/3, 0) in fractional reciprocal coordinates, respectively] along this band structure path and a doubly orbital-degenerate Te *p* VBM. Reduced energy differences between adjacent bands near the Fermi level result in increased effective band degeneracy, thus yielding a higher effective DOS in Sc_2_Si_2_Te_6_. As a result of the differing locations of the band extrema in Sb_2_Si_2_Te_6_ (both VBM and CBM at *Z*) and Sc_2_Si_2_Te_6_ (VBM at Γ and CBM at Σ), there is a significant difference in their valley (*k*-point) degeneracy. This point is demonstrated by the Fermi surfaces plotted using the IFermi package^47^ in Figure 3. Due to the symmetry of the Brillouin zone, there are two-half electron (hole) pockets at the *Z* point for Sb_2_Si_2_Te_6_, and thus the *k*-point degeneracy is 1 for both VBM and CBM (Figure 3a). For Sc_2_Si_2_Te_6_, the VBM is at Γ, and an isolated full-hole pocket is generated in the center of the Brillouin zone, again corresponding to a *k*-point degeneracy of 1. Meanwhile, six relatively flat electron pockets appear close to the edges of the Brillouin zone for the CBM (Figure 3b,c). These electron pockets show significant anisotropy, being mostly flat along the *z* (cross-plane) direction but relatively disperse along the horizontal (in-plane) direction. This is further confirmed by the flat conduction band along the band structure path between the Σ, Σ′, and Σ″ degenerate *k*-points located within the same anisotropic band valley and labeled in Figure 3c at (1/2, −1/6, 1/6), (1/3, −1/3, 0), and (1/6, −1/2, −1/6) in fractional reciprocal coordinates, respectively, shown in Figure S3. Due to the rhombohedral crystal symmetry (with hexagonal symmetry within each ABC-stacked layer) and the location of these band edge *k*-points (valleys) within the Brillouin zone rather than at an edge or vertex, each anisotropic valley is 6-fold degenerate, as shown in Figure 3.

The origin of this anisotropy and degeneracy enhancement can be further elucidated by analyzing the band orbital character and structural symmetry. As shown in the band structure and *lm*-decomposed orbital-projected density of states plots in Figures S4 and S5, the orbital character of the conduction band in Sc_2_Si_2_Te_6_ shows significant *k*-point dependence. At the degenerate conduction band minima (Σ/Σ′/Σ″), equal contributions of Sc *d*_*xy*_, *d*_*yz*_, *d*_*xz*_, and *d*_*x*^2^–*y*^2^_ (but no *d*_*z*_^2^) orbitals are obtained, along with some minor Si *p*_*z*_ contributions, yielding the hybridized orbital state shown in Figure 2c. This orbital combination maximizes the bonding interactions between unoccupied Sc *d* and Si *p* states, while minimizing antibonding Sc–Te interactions for the Σ/Σ′/Σ″ *k*-points at the CBM edge, as indicated by the Crystal Orbital Hamilton Population (COHP) plots in Figure S6. The real space directions corresponding to these *k*-points are shown in Figure S7, where we see that they are coplanar, corresponding to in-layer and slightly off-layer for the phase factor direction in Sc_2_Si_2_Te_6_. The weak energy dependence on the phase factor along the interlayer direction for the Sc *d* CBM within this *k*-point range is attributed to the lack of *d*_*z*_^2^ orbital character for these states. This weak interlayer dispersion combined with strong intralayer dispersion yields the highly anisotropic CBM electron pockets shown in Figure 3. Overall, we can see that this anisotropy and degeneracy enhancement for the CBM in Sc_2_Si_2_Te_6_ arises as a consequence of the hexagonal layer symmetry with ABC stacking and the hybridized Sc *d* and Si *p* orbital symmetries.

Combined, these factors dramatically enhance the DOS effective mass *m*_DOS_^*^ in Sc_2_Si_2_Te_6_, while retaining a relatively small conductivity mass *m*_σ_^*^. The single-band carrier effective masses for both compositions are summarized in Table 2. Sc_2_Si_2_Te_6_ displays larger and more anisotropic effective masses for both electrons and holes compared to Sb_2_Si_2_Te_6_ as expected, which is also represented by the smaller Fermi velocities of Sc_2_Si_2_Te_6_ compared to Sb_2_Si_2_Te_6_ (Figure 3). Therefore, the heightened band anisotropy and degeneracy is anticipated to result in a significantly higher power factor for Sc_2_Si_2_Te_6_.

**Table 2:** Hole and Electron Effective Masses (*m*_e_) of Sb_2_Si_2_Te_6_ and Sc_2_Si_2_Te_6_ Were Calculated Using Parabolic Fitting in Sumo with the HSE06 + SOC Functional^a^

system	hole (*m*_e_)	electron (*m*_e_)
Sb_2_Si_2_Te_6_	0.17 (Z–X) 0.28 (Z–B) 0.28 (Z–Q)	0.29 (Z–X) 0.09 (Z–B) 0.09 (Z–Q)
Sc_2_Si_2_Te_6_	0.32 (L–B_1_) 0.33 (Γ–X) 3.67 (Γ–B)	0.43 (Σ–B_1_) 0.32 (Σ–Γ)

a The corresponding *k*-point path is given in parentheses.

### Band Alignment

**Figure 4:**
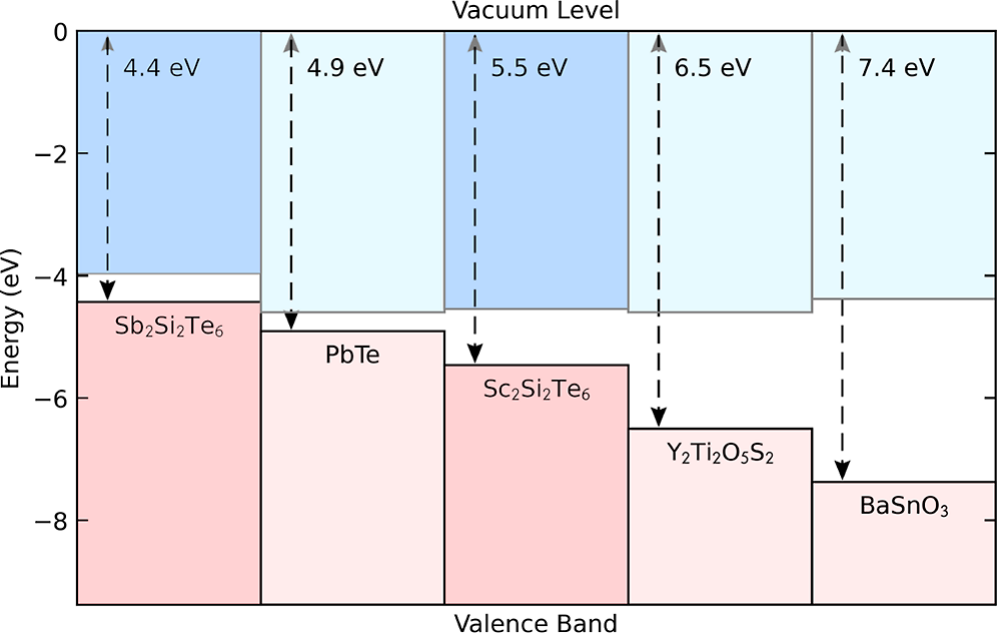
Band alignment of Sb_2_Si_2_Te_6_ and Sc_2_Si_2_Te_6_ calculated in this study and compared to the band alignments of bipolar PbTe,^49^ n-type Y_2_Ti_2_O_5_S_2_,^50^ and BaSnO_3_.^51^ IP is annotated.

The band alignment of Sb_2_Si_2_Te_6_ and Sc_2_Si_2_Te_6_ is calculated using the core-level alignment approach (see Supporting Information for more details).^48^ The results are depicted in Figure 4 together with the band alignment of several other high-performance thermoelectric materials. The electron affinities (EA) and ionization potentials (IP) can provide an indication of the propensity to p- or n-type dopability. Compared to bipolar-dopable PbTe, the IP of Sb_2_Si_2_Te_6_ is smaller (due to the antibonding Sb s–Te p interaction at the VBM), while that of Sc_2_Si_2_Te_6_ is larger, indicating that the formation of p-type defects may be more likely for Sb_2_Si_2_Te_6_. Indeed, Sb_2_Si_2_Te_6_ exhibits strong p-type behavior experimentally, which is attributed to Sb vacancies.^4^ The EA of Sc_2_Si_2_Te_6_ is analogous to that of PbTe, n-type Y_2_Ti_2_O_5_S_2_ and BaSnO_3_, while that of Sb_2_Si_2_Te_6_ is smaller, suggesting that Sc_2_Si_2_Te_6_ may favor n-type doping.

### Electronic Transport Properties

**Figure 5:**
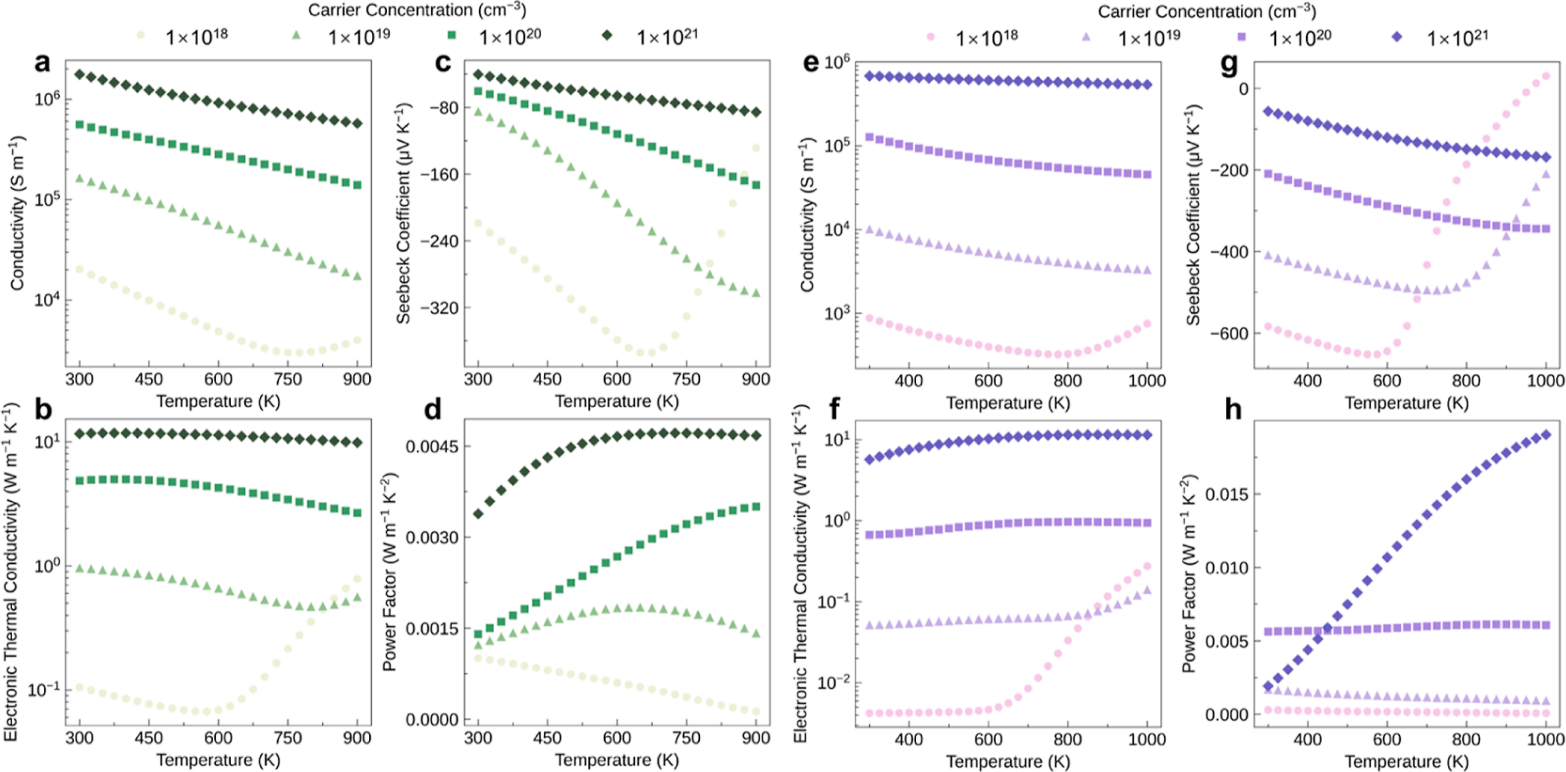
Calculated electronic transport properties as a function of temperature for n-type (a–d) Sb_2_Si_2_Te_6_ and (e–h) Sc_2_Si_2_Te_6_ with four different carrier concentrations. Sb_2_Si_2_Te_6_ and Sc_2_Si_2_Te_6_ have demonstrated thermal stability up to 920 and 1023 K,^4,16^ respectively, and so these ranges are used in our analysis.

**Figure 6:**
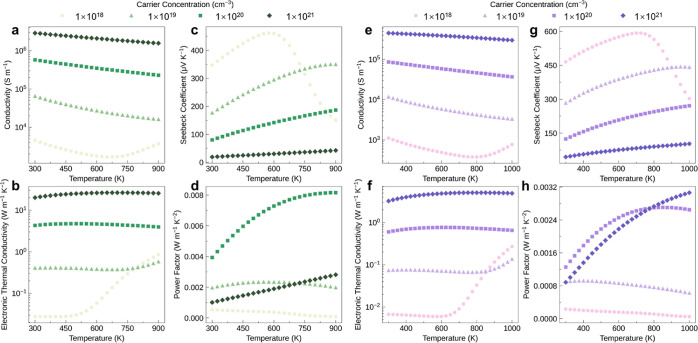
Calculated electronic transport properties as a function of temperature for p-type (a–d) Sb_2_Si_2_Te_6_ and (e–h) Sc_2_Si_2_Te_6_ with four different carrier concentrations.

Using the calculated electronic (Figure 2), phonon (Figure S12), and electron–phonon properties from density functional theory (DFT), the carrier mobilities were computed using the Boltzmann transport equations as implemented in the AMSET package (see Computational Methods and Supporting Information).^52^ The individual scattering mechanisms are plotted as functions of temperature, carrier concentration, and electronic energy in Figures S8 and S9. As expected, the dominant mobility-limiting scattering mechanism varies between polar-optical phonon (POP), ionized impurity (IMP), or acoustic deformation potential (ADP) scattering, depending on the doping/carrier concentration and temperature. Under temperature and doping concentrations that maximize *ZT* (shown later), carrier-phonon scattering dominates for Sb_2_Si_2_Te_6_—primarily due to the lower energy phonon modes from the heavier Sb atoms (Figure S12)—with POP scattering dominant under p-type doping and ADP scattering under n-type doping. For Sc_2_Si_2_Te_6_ on the other hand, IMP scattering dominates under *ZT*-optimized conditions. The resultant electronic transport properties for n-type and p-type doping across a range of carrier concentrations and temperatures are shown in Figures 5 and 6, respectively. As expected, we witness decreases in electrical conductivity with increasing temperature; however, the effect is relatively minor for high carrier concentrations due to the dominance of (mostly) temperature-independent impurity scattering over carrier-phonon scattering in these regimes. For n-type Sb_2_Si_2_Te_6_, the temperature dependence is stronger due to greater carrier-phonon interactions for this case. Overall, Sb_2_Si_2_Te_6_ exhibits higher electrical conductivity than Sc_2_Si_2_Te_6_ under both n and (particularly) p-type conditions due to the lower carrier effective masses (Figure 2 and Table 2) and scattering rates (Figures S8 and S9). The enhanced band degeneracy in Sc_2_Si_2_Te_6_ may also increase the rate of intervalley scattering, contributing to a decrease in carrier mobility.^32^ As expected from the Wiedemann–Franz law (κ_e_ = *L*σ*T*, where *L* is the Lorentz number and *T* is temperature), the trends in electronic thermal conductivity (κ_e_) mostly follow those of electrical conductivity (σ).

The Seebeck coefficient (*S*) reflects the voltage generated in response to a temperature gradient, and its sign depends on the dominant charge carrier type (positive for holes, negative for electrons). In general, the Seebeck coefficient can be expressed as
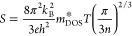
1where *k*_B_ is the Boltzmann constant, *e* represents the elementary charge, *h* denotes Planck’s constant, *n* is the carrier concentration, and *m*_DOS_^*^ represents the DOS effective mass.^53^ Thus, as witnessed in Figures 5 and 6, the Seebeck coefficient typically exhibits opposite dependence on temperature and carrier concentration compared to the electrical conductivity, decreasing with carrier concentration and increasing with temperature due to their effects on carrier diffusion (and corresponding voltage) across a temperature gradient in a material. For both n and p-type conditions, we see that the Seebeck coefficient in Sc_2_Si_2_Te_6_ consistently exceeds that of Sb_2_Si_2_Te_6_, showing the opposite behavior to the electrical conductivity. In particular, n-type Sc_2_Si_2_Te_6_ retains a large Seebeck coefficient (*S* > 300 μV/K) even at relatively high carrier concentrations (*n* = 10^20^ cm^–3^) and electrical conductivities (σ ∼ 10^5^ S/m). According to eq 1, for a given carrier concentration and temperature, the Seebeck coefficient is primarily determined by the DOS effective mass—which can be approximated by the equation  .^38^ Here, *N*_v_ is the number of degenerate valleys in the electronic band structure, and m* is the effective mass within a single valley. As discussed previously, Sc_2_Si_2_Te_6_ exhibits increased band degeneracy (6 and 2 for n and p-type, respectively, vs 1 for both carrier types in Sb_2_Si_2_Te_6_) and carrier effective masses (Table 2) compared to Sb_2_Si_2_Te_6_, elucidating the origins of increased Seebeck coefficients for Sc_2_Si_2_Te_6_. The calculated electronic transport properties, broken down into the in-plane and cross-plane directions for these layered materials, are provided in the Supporting Information, with Sc_2_Si_2_Te_6_ showing pronounced anisotropy (discussed further below). Additionally, the nonmonotonic behavior for the Seebeck coefficients at low carrier concentrations is due to the bipolar conduction effect, with further discussion provided in the Supporting Information.

The power factor (PF = *S*^2^σ) is a key parameter in thermoelectric performance (*ZT* ∝ PF), which can have a complex dependence on carrier concentration and temperature due to their competing effects on *S* and σ. As a result, a balanced carrier concentration, striking an equilibrium between the Seebeck coefficient and electrical conductivity, is necessary to maximize PF. The optimal PF is higher for p-type (8.16 mW m^–1^ K^–2^) rather than n-type (4.67 mW m^–1^ K^–2^) Sb_2_Si_2_Te_6_ (Figures 5 and 6). In contrast, for Sc_2_Si_2_Te_6_, the highest PF of n-type is 19 mW m^–1^ K^–2^, which significantly exceeds the value of 3.06 mW m^–1^ K^–2^ for p-type doping (Figures 5 and 6). Moreover, this optimal PF for n-type Sc_2_Si_2_Te_6_ markedly exceeds the PF predicted for various layered thermoelectric materials, such as BiCuOSe (∼1.71 mW m^–1^ K^–2^),^41^ while it still lags behind that predicted for monolayer SnSe (∼28 mW m^–1^ K^–2^).^54^ This ultrahigh PF in n-type Sc_2_Si_2_Te_6_ primarily originates from the enhanced conduction band degeneracy and anisotropy, as discussed above. Enhancing the PF by band engineering has also been reported in other materials, such as PbTe, which achieves a band degeneracy of 16 by tuning the doping and composition.^32^

### Thermal Transport Properties

**Figure 7:**
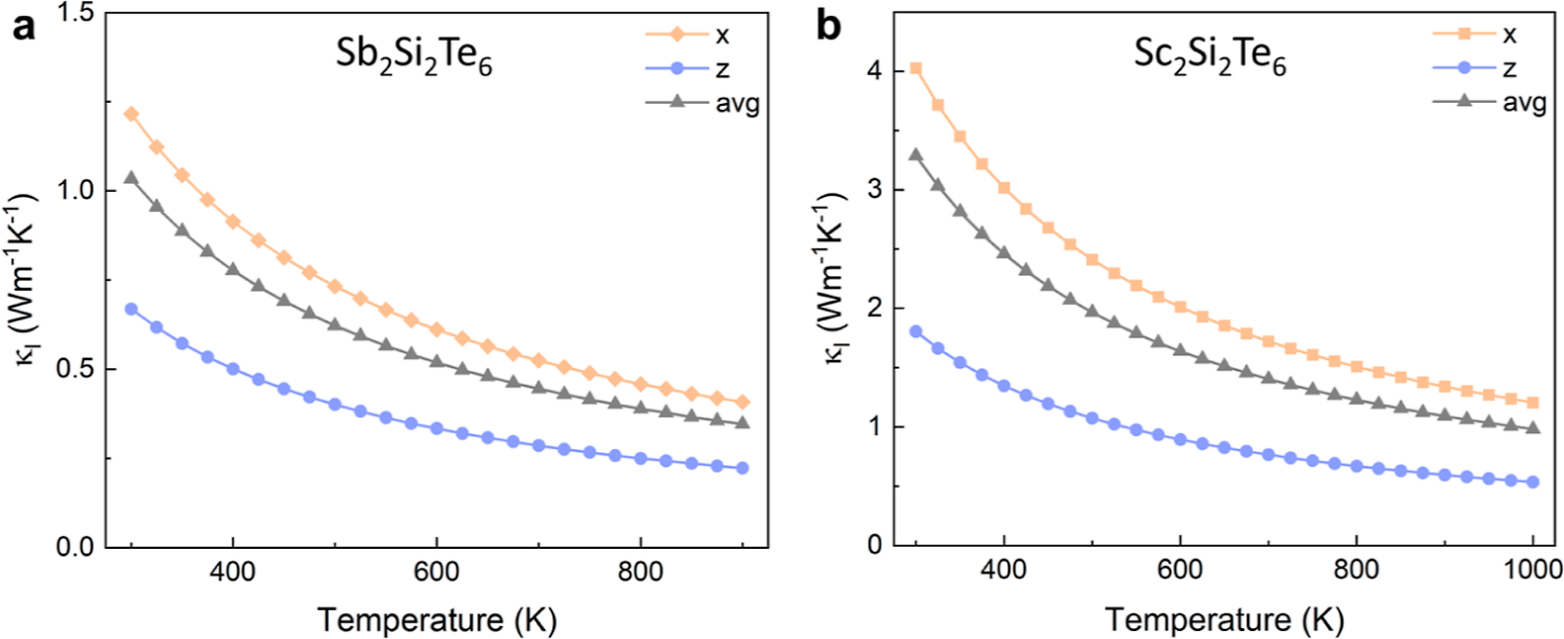
Calculated lattice thermal conductivity κ_l_ of (a) Sb_2_Si_2_Te_6_ and (b) Sc_2_Si_2_Te_6_ as a function of temperature. Sb_2_Si_2_Te_6_ and Sc_2_Si_2_Te_6_ have demonstrated thermal stability up to 920 and 1023 K,^4,16^ respectively, and so these ranges are used in our analysis.

The calculated lattice thermal conductivity κ_l_ of both compounds is shown in Figure 7. The lattice thermal conductivity decreases with temperature increasing due to greater phonon–phonon scattering at higher temperatures. The lattice thermal conductivity of both compounds is found to be relatively low as expected, particularly in the interlayer (*z*) direction, with Sc_2_Si_2_Te_6_ exhibiting higher lattice thermal conductivity values than Sb_2_Si_2_Te_6_ (by a factor of ∼3×). This behavior can be rationalized through the calculated phonon dispersions, which are provided in Figure S12. Given their structural and compositional similarity, the phonon dispersions are relatively similar, with many flat bands present—indicating low phonon group velocities and the likelihood of low lattice thermal conductivity—and the heavy Te atoms dominating the low-energy optical and acoustic modes in both cases. Due to the comparable mass of Sb (121.76 au) and Te (127.60 au) in Sb_2_Si_2_Te_6_ (cf. 44.96 au for Sc), a greater density of phonon bands is witnessed in the low-frequency range (0–5 THz) compared to Sb_2_Si_2_Te_6_, leading to increased phonon scattering and thus reduced lattice thermal conductivity, as witnessed in Figure 7.

The room temperature lattice thermal conductivity (1.03 W m^–1^ K^–1^) of Sb_2_Si_2_Te_6_ closely aligns with the experimental value (1.0 W m^–1^ K^–1^)^4^ and is also comparable to that of other layered thermoelectric materials, such as BiCuOSe (1.0 W m^–1^ K^–1^)^41^ and Bi_2_O_2_Se (1.1 W m^–1^ K^–1^).^55^ Importantly, the lattice thermal conductivity is highly anisotropic in both Sb_2_Si_2_Te_6_ and Sc_2_Si_2_Te_6_, resulting in average κ_*x*,*y*_/κ_*z*_ ratios of 1.82 and 2.24, respectively, at 300 K. The anisotropic nature of lattice thermal conductivity can be attributed to the difference between relatively weak (van der Waals) interlayer interactions along the cross-plane direction and strong (covalent) intralayer interactions along the in-plane direction. Further analysis of the atomistic origins of thermal transport behavior in these layered compounds was performed by querying the frequency-dependent phonon group velocities, lifetimes, and cumulative lattice thermal conductivity, as provided in the Supporting Information (Figures S13 and S14). These analyses further confirmed the role of Sb/Te mass similarity in the reduced lattice thermal conductivity of Sb_2_Si_2_Te_6_, leading to enhanced phonon–phonon scattering and thus reduced phonon lifetimes, rather than any major differences in group velocities.

### Thermoelectric Figure of Merit

**Figure 8:**
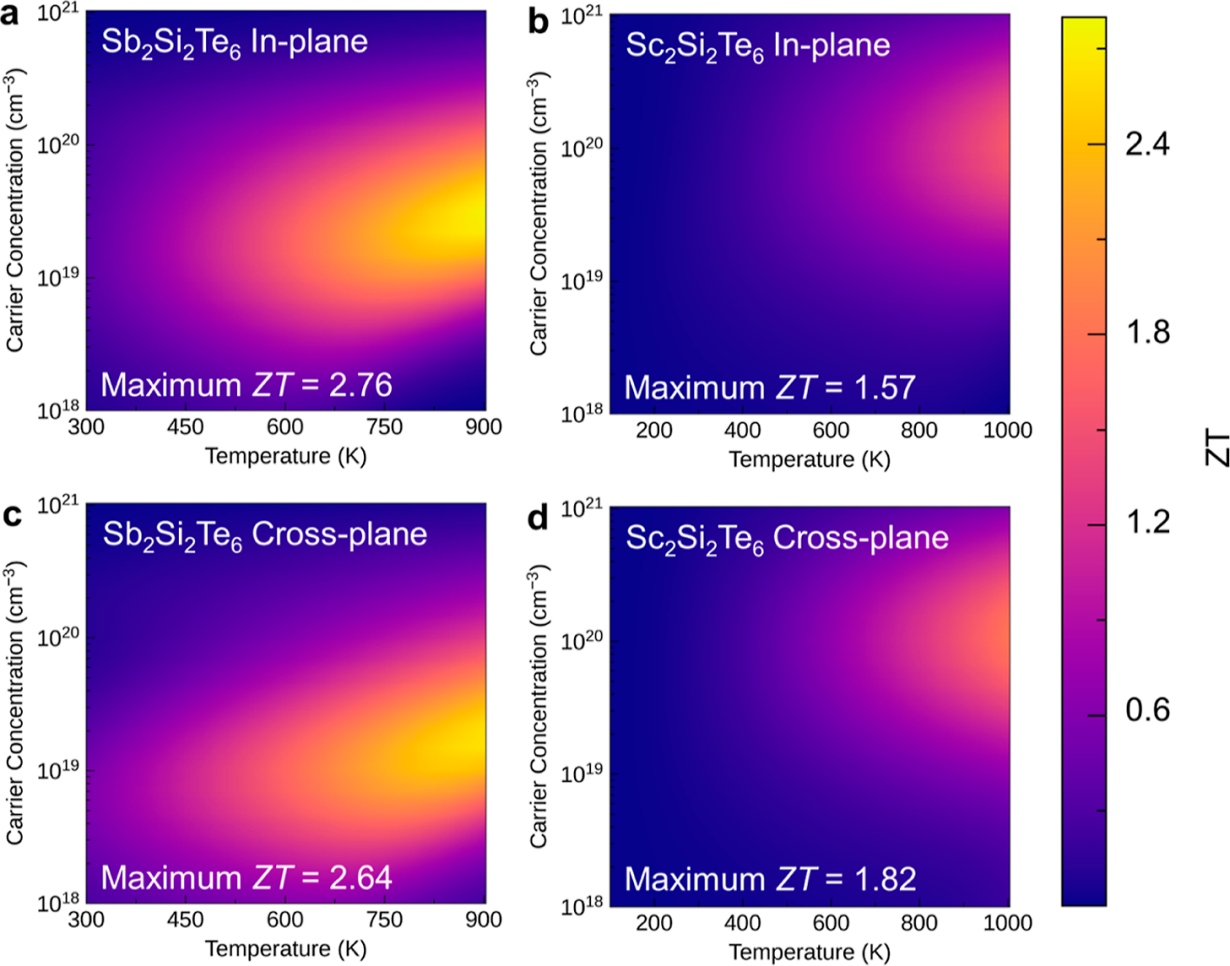
Predicted thermoelectric figure of merit *ZT* of p-type (a,c) Sb_2_Si_2_Te_6_ and (b,d) Sc_2_Si_2_Te_6_ against temperature and carrier concentration along the in-plane and cross-plane directions. The lightest colors indicate the highest *ZT*.

**Figure 9:**
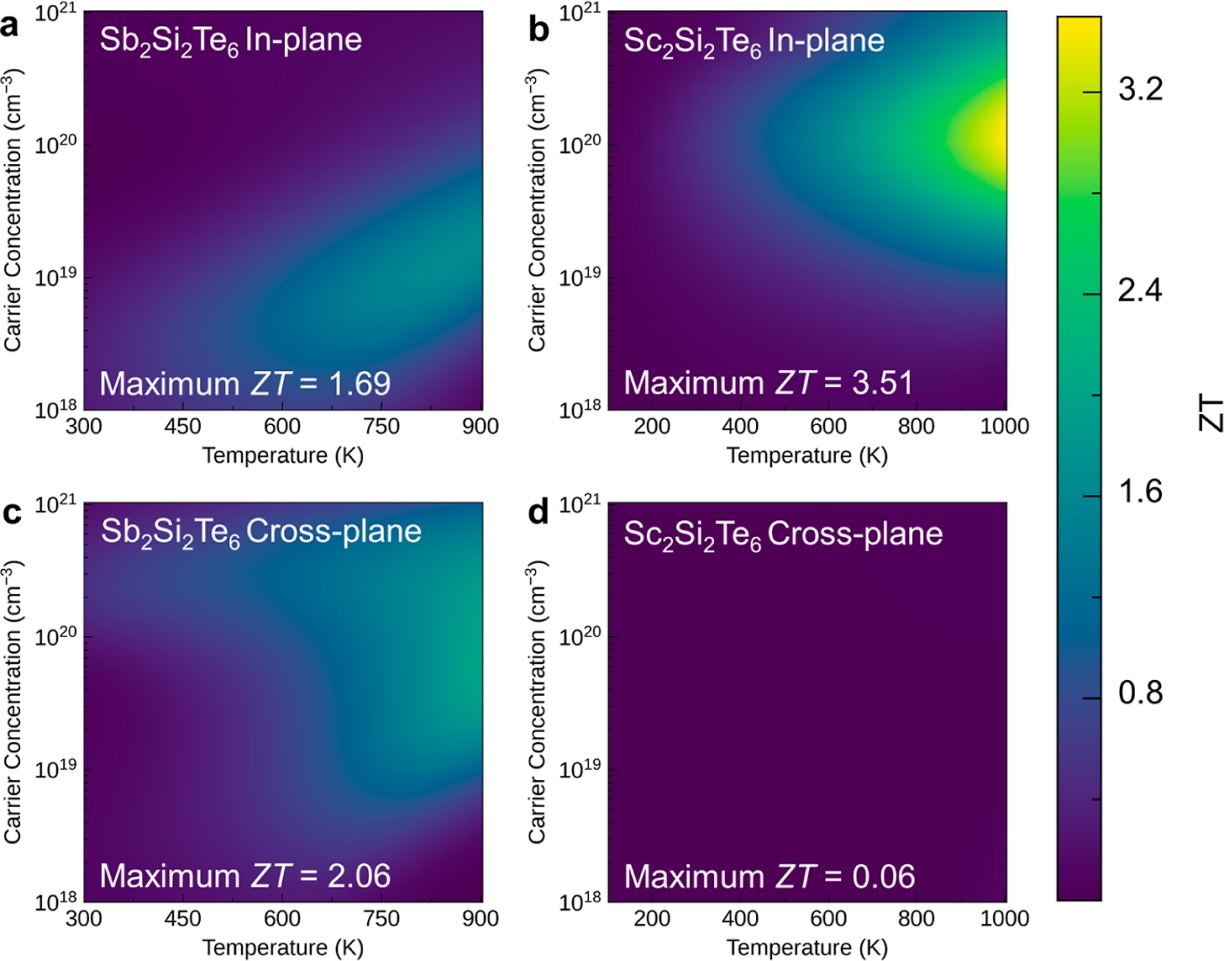
Predicted thermoelectric figure of merit *ZT* of n-type (a,c) Sb_2_Si_2_Te_6_ and (b,d) Sc_2_Si_2_Te_6_ against temperature and carrier concentration along the in-plane and cross-plane directions. The lightest colors indicate the highest *ZT*.

With these electrical and thermal transport properties, we can calculate the theoretical dimensionless figure of merit *ZT* for Sb_2_Si_2_Te_6_ and Sc_2_Si_2_Te_6_ as functions of temperature and carrier concentration (Figures 8 and 9). It is found that for Sb_2_Si_2_Te_6_, the p-type system achieves a superior *ZT* compared to the n-type system, while the results are opposite in Sc_2_Si_2_Te_6_. For p-type doping, Sb_2_Si_2_Te_6_ consistently exhibits higher *ZT* than Sc_2_Si_2_Te_6_ regardless of the transport direction, which can be attributed to the lower lattice thermal conductivity (Figure 7) and higher electrical conductivity (Figure 6). However, for n-type doping, we find an extremely large calculated *ZT* of 3.51 at 1000 K for the in-plane direction in Sc_2_Si_2_Te_6_, greatly surpassing the optimal *ZT* observed (1.69–2.06) for n-type Sb_2_Si_2_Te_6_. The exceptional *ZT* observed for n-type Sc_2_Si_2_Te_6_ originates from the dramatically enhanced Seebeck coefficient and PF (Table 4) caused by high band degeneracy and anisotropy, as discussed above, in combination with the low lattice thermal conductivity (Figure 7). This value surpasses that of typical layered thermoelectric materials, such as p-type SnSe (measured ZT of ∼2.6 at 923 K) and BiCuOSe (predicted *ZT* of 1.32 at 1000 K).^7,56^ Similar predictions of high *ZT* through high Seebeck coefficient and PF have also been observed in other materials, such as Na_2_TlSb (*ZT* ∼ 4.81) and InBrSe (*ZT* ∼ 5.12).^57,58^ We note that while the in-plane *ZT* is extremely high for n-type Sc_2_Si_2_Te_6_, the cross-plane *ZT* is very low (0.06 at 1000 K, the *ZT* with color scale of n-type Sc_2_Si_2_Te_6_ along the cross-plane direction as shown in Figure S15), which can be attributed to the low cross-plane n-type conductivity (Figure S10)—itself a consequence of the relatively localized Sc d orbitals in the Sc_2_Si_2_Te_6_ CBM, giving weak interlayer electronic interactions. Overall, these results suggest that Sc_2_Si_2_Te_6_ holds promise as an excellent thermoelectric material for applications in the moderate-to-high temperature range and also demonstrate the power of band engineering for enhancing thermoelectric performance within compound families. The calculated optimal *ZT* and associated carrier concentrations, PF, electronic thermal conductivity, and lattice thermal conductivity for Sb_2_Si_2_Te_6_ and Sc_2_Si_2_Te_6_ are summarized in Tables 3 and 4.

**Table 3:** Predicted Maximum *ZT* in Sb_2_Si_2_Te_6_ along the In-Plane and Cross-Plane Directions Together with Corresponding Charge Carrier Concentration (*n*), Power Factor (PF), Electronic Thermal Conductivity (κ_e_), and Lattice Thermal Conductivity (κ_l_)

system	direction	*ZT*	*n* (cm^–3^)	PF (μW m^–^^1^ K^–^^2^)	κ_e_ (W m^–^^1^ K^–^^1^)	κ_l_ (W m^–^^1^ K^–^^1^)
n-type	*xy*	1.69	1.47 × 10^19^	2160	0.70	0.42
	*z*	2.06	6.81 × 10^19^	3520	1.32	0.22
p-type	*xy*	2.76	3.16 × 10^19^	5310	1.32	0.41
	*z*	2.64	1.47 × 10^19^	2120	0.5	0.22

**Table 4:** Predicted Maximum *ZT* in Sc_2_Si_2_Te_6_ along the In-Plane and Cross-Plane Directions Together with Corresponding Charge Carrier Concentrations (*n*), Power Factor (PF), Electronic Thermal Conductivity (κ_e_), and Lattice Thermal Conductivity (κ_l_)

system	direction	*ZT*	*n* (cm^–3^)	PF (μW m^–^^1^ K^–^^2^)	κ_e_ (W m^–^^1^ K^–^^1^)	κ_l_ (W m^–^^1^ K^–^^1^)
n-type	*xy*	3.51	1.16 × 10^20^	9110	1.40	1.21
	*z*	0.06	4.64 × 10^20^	30.2	0.014	0.54
p-type	*xy*	1.57	1.06 × 10^20^	3150	0.80	1.21
	*z*	1.82	1.09 × 10^20^	1640	0.37	0.54

We note that the theoretically predicted maximum *ZT* for p-type Sb_2_Si_2_Te_6_ exceeds the experimentally reported result (∼1.08 at 823 K, increased to ∼1.65 when incorporated in a cellular nanostructure with Si_2_Te_3_).^4^ Notably, the calculated lattice thermal conductivity of Sb_2_Si_2_Te_6_ matches well with the experimental measurements, suggesting that the primary origin of reduced *ZT* in experiment is due to additional scattering mechanisms and nonidealities in the electronic properties. This discrepancy can be explained by the following two aspects. On the one hand, in experiment, thermal radiation,^59^ air-induced thermal convection,^60^ impurity phases and the effect of grain boundary scattering on carrier mobility^61^ can lead to degradation of thermoelectric performance—effects which are not included in our theoretical model. On the other hand, this experimental value corresponds to a single carrier concentration (measured to be ∼5.6 × 10^19^ cm^–3^ at room temperature) which may vary with temperature, but we calculate the thermoelectric properties over a range of temperatures and carrier concentrations. Indeed, as expected, we find a high sensitivity of the overall *ZT* to the carrier concentration (Figure 8), with different optimal concentrations for the in-plane and cross-plane directions. Thus, given the unoptimized experimental carrier concentration and potential additional factors not accounted for in our model, a lower experimental figure of merit is expected. Our simulations therefore reflect an idealized model which can help guide experimental efforts in further improving the thermoelectric performance of this material. In particular, the anisotropy in thermoelectric properties indicates that controlled-orientation single crystals (with optimized carrier concentrations) are expected to achieve significantly higher *ZT* values than polycrystalline samples for these layered compounds.

## Summary

In conclusion, our investigation systematically explored the intrinsic thermoelectric properties of Sb_2_Si_2_Te_6_ and Sc_2_Si_2_Te_6_, employing a combination of semiclassical Boltzmann transport theory and first-principles calculations. While both layered compounds exhibit low intrinsic lattice thermal conductivities, the low mass difference between Sb and Te results in greater phonon–phonon scattering and shorter phonon lifetimes in Sb_2_Si_2_Te_6_, yielding very low lattice thermal conductivity. We find substantial differences in the electronic structures of these two compounds due to the different orbital characters of the A-site cations. Specifically, Sb_2_Si_2_Te_6_ manifests a small, direct band gap, while Sc_2_Si_2_Te_6_ exhibits a larger, indirect band gap. The combination of crystal (ABC-stacked hexagonal layers) and orbital (hybridized Sc *d* and Si *p*) symmetries at the Sc_2_Si_2_Te_6_ CBM gives rise to nonhigh-symmetry band-edge *k*-points, dramatically enhancing the overall band degeneracy and anisotropy. Combined with good electron mobility in the in-plane direction, this yields an ultrahigh power factor (19 mW m^–1^ K^–2^) and thus, along with the low lattice thermal conductivity, a large predicted *ZT* of 3.51 for n-type Sc_2_Si_2_Te_6_ (compared to the optimal *ZT* of 2.76 for p-type Sb_2_Si_2_Te_6_). This enhanced *ZT* arises despite the ∼3× increase in lattice thermal conductivity upon substituting Sb with Sc, as the band degeneracy and anisotropy enhancements of the PF overcomes this loss. We note that this optimal *ZT* requires a relatively high charge carrier concentration (*n* ∼ 10^20^ cm^–3^), so understanding the defect (dopant) chemistry and possible engineering strategies will be key to realizing this performance experimentally.^62,63^ This study demonstrates the potential of Sc_2_Si_2_Te_6_ for thermoelectric applications and, more broadly, the power of band engineering as a route to designing high-performance thermoelectric materials.

## Computational Methods

The calculations were performed within DFT as implemented in the Vienna ab initio simulation package (VASP) code.^64,65^ The projector augmented wave (PAW) pseudopotential method was employed to account for the interaction between core and valence electrons.^66,67^ A plane-wave kinetic energy cutoff of 400 eV and a 6 × 6 × 6 Γ-centered *k*-point grid was used for a 10-atom primitive cell, ensuring convergence of total energy within 1 meV/atom using vaspup2.0.^70^ The cutoff was increased to 520 eV during geometry optimizations to avoid Pulay stress. To account for van der Waals dispersion interactions, the D3 correction of Grimme et al. was applied.^68,69^ The structures were fully relaxed using the Heyd–Scuseria–Ernzerhof (HSE06) hybrid DFT functional,^71,72^ except for lattice dynamics calculations (ionic contribution to the dielectric constant, POP frequency, and phonon force constants) for which the Perdew–Burke–Ernzerhof generalized gradient approximation (GGA) functional for solids (PBEsol) was used.^73,74^ There are no constraints imposed on the unit cell shape or size, and relaxation continued until the change in maximum force on the ions and electronic free energy did not exceed 0.0005 eV/Å and 1 × 10^–8^ eV, respectively. Electronic structure and high-frequency dielectric constant calculations used HSE06 including spin–orbit coupling (SOC) effects.

The doped defect calculation software^45^ was used to collate all competing phases in the Sc–Si–Te chemical space within 0.1 eV/atom of the convex hull according to the Materials Project database—which were then re-relaxed and calculated using HSE06 + SOC, parsed the calculation outputs, and plotted the calculated chemical stability region (chemical potential ranges) for Sc_2_Si_2_Te_6_.

The AMSET software package was employed to calculate the electronic transport properties.^52^ Typically, the Boltzmann transport equations are solved using the constant relaxation-time approximation (CRTA), which usually results in the overestimation of *ZT*s.^52,75^ Instead, AMSET improves upon the CRTA by using the momentum relaxation-time approximation (MRTA) to explicitly calculate scattering rates for the individual electronic states within the Born approximation. AMSET offers insights into the contributions to the transport properties from four types of scattering: POP scattering, ADP scattering, IMP scattering, and piezoelectric (PIE) scattering. These contributions are integrated into the *ZT* equation via the electrical conductivity and electronic thermal conductivity. The PIE constant was calculated to be negligible for these materials, and so PIE scattering is not included in the analysis. The Seebeck coefficient is also calculated by AMSET but is not influenced by scattering.

The material parameters required as input for AMSET, such as high-frequency and static dielectric constants, elastic constants, phonon frequencies, and deformation potentials, were obtained by first-principles calculations. Ionic dielectric constant contributions and the Γ-point phonon frequencies and dipole moments required to calculate the polar-optic phonon frequency in AMSET were acquired using the finite-displacement method with the PBEsol functional. Deformation potential and high-frequency dielectric constant were determined using HSE06 + SOC. These data are available in Table S1. The transport properties have been converged with respect to the interpolation factor, as shown in Figure S16.

Lattice dynamics calculations were performed using the finite-displacement method within the Phonopy package.^76^ Convergence of the phonon dispersion with the supercell size used for second-order force constants (FCs) was explicitly checked, as illustrated in Figure S17. Second-order FCs were computed using a 3 × 3 × 1 supercell derived from the 30-atom conventional cell, employing the PBEsol functional.^77^ Atom contributions to the lattice dynamics were determined from the atom-projected phonon density of states (PDOS), calculated through Fourier interpolation. The lattice thermal conductivity was calculated using the Phono3py package,^78^ which employs a supercell method to calculate the third-order FCs with the PBEsol functional. A 2 × 2 × 1 supercell based on the conventional cell was used to calculate the third-order FCs. The lattice thermal conductivity was determined by solving the phonon Boltzmann transport equation within the single-mode relaxation-time approximation. The convergence of *q*-point mesh density was verified, and a 13 × 13 × 13 mesh was selected, as illustrated in Figure S18. Plots of the electronic, phonon, and thermoelectric properties were generated using sumo,^79^ IFermi,^47^ and ThermoParser.^80^

## Data Availability

Calculation data and parsed outputs are provided in an openly available online repository at http://doi.org/10.5281/zenodo.11165391.

## Supporting Information Available

Competing phases analysis, additional electronic analysis, scattering rates analysis, additional electronic transport properties analysis, phonon dispersion and phonon DOS, additional thermal transport properties analysis, predicted thermoelectric figure of merit *ZT*, convergence of the electronic transport properties with respect to the interpolation factor, phonon supercell convergence testing, convergence of κ_l_ with *q*-point sampling mesh, and band alignment calculations (PDF)
