# Supplementary material for: Band Degeneracy and Anisotropy Enhances Thermoelectric Performance from Sb2Si2Te6 to Sc2Si2Te6
Source: J Am Chem Soc. 2024 Jun 18;146(26):17679–90. doi: 10.1021/jacs.4c01838 (PMC11228999; doi:10.1021/jacs.4c01838)
Supplement: Supplementary file 1 — ja4c01838_si_001.pdf [file ja4c01838_si_001.pdf]

Supplementary Information For:

Band Degeneracy and Anisotropy Enhances Thermoelectric Performance from $Sb_2Si_2Te_6$ to $Sc_2Si_2Te_6$

Wenzhen Dou[a, b, f], Kieran B. Spooner[b, c, e], Seán R. Kavanagh[b, d, e,*], MiaoZhou[a, f, g,*] and David O. Scanlon[b, c, e,*]

[a] School of Physics, Beihang University, Beijing 100191, China

[b] Department of Chemistry, University College London, London WC1H 0AJ, United Kingdom

[c] School of Chemistry, University of Birmingham, Birmingham B15 2TT, United Kingdom

[d] John A. Paulson School of Engineering and Applied Sciences, Harvard University, Cambridge MA 02138, USA

[e] Thomas Young Centre, University College London, London WC1E 6BT, United Kingdom

[f] Hangzhou International Innovation Institute, Beihang University, Hangzhou 311115, China

[g] Tianmushan Laboratory, Hangzhou 310023, China

***E-mail:** skavanagh@seas.harvard.edu (S.R.K.); mzhou@buaa.edu.cn (M.Z.); d.o.scanlon@bham.ac.uk (D.O.S.)

AMSET settings

Table S1: The *k*-point meshes used in the zero-weighted density of states (DOS), the interpolated DOS used in AMSET, finite displacement method and optics calculations for $Sb_2Si_2Te_6$ and $Sc_2Si_2Te_6$. The high-frequency dielectric constant was calculated from the optics calculation, the ionic dielectric constant, elastic constant, piezoelectric constant (of 0) and polar optical phonon frequency were calculated from the finite displacement method. The static dielectric constant is the sum of the high-frequency and ionic dielectric constants.

Calculation	Zero-weighted DOS	Interpolated DOS	Finite displacement	Optics
k-point mesh	10 × 10 × 10	51 × 51 × 51	8 × 8 × 8	6 × 6 × 6

These are the inputs for AMSET in $Sb_2Si_2Te_6$

$$\text{High-frequency dielectric constant } (\epsilon) = \begin{bmatrix} 18.94 & 0 & 0 \\ 0 & 18.94 & 0 \\ 0 & 0 & 16.09 \end{bmatrix}$$

$$\text{Static dielectric constant } (\epsilon) = \begin{bmatrix} 44.10 & 0 & 0 \\ 0 & 44.10 & 0 \\ 0 & 0 & 18.29 \end{bmatrix}$$

$$\text{Elastic constant (GPa)} = \begin{bmatrix} 75.6 & 21.7 & 24.8 & 0 & 15.7 & 0 \\ 21.7 & 75.6 & 24.8 & 0 & -15.7 & 0 \\ 24.8 & 24.8 & 49.9 & 0 & 0 & 0 \\ 0 & 0 & 0 & 27.4 & 0 & -15.7 \\ 15.6 & -15.6 & 0 & 0 & 27.0 & 0 \\ 0 & 0 & 0 & -15.7 & 0 & 27.0 \end{bmatrix}$$

Polar optical phonon frequency (THz) = 4.47

These are the inputs for AMSET in $Sc_2Si_2Te_6$

$$\text{High-frequency dielectric constant } (\epsilon) = \begin{bmatrix} 7.89 & 0 & 0 \\ 0 & 7.89 & 0 \\ 0 & 0 & 5.80 \end{bmatrix}$$

$$\text{Static dielectric constant } (\epsilon) = \begin{bmatrix} 13.75 & 0 & 0 \\ 0 & 13.75 & 0 \\ 0 & 0 & 6.31 \end{bmatrix}$$

$$\text{Elastic constant (GPa)} = \begin{bmatrix} 86.7 & 20.4 & 20.1 & 0 & 9.7 & 0 \\ 20.4 & 86.7 & 20.1 & 0 & -9.7 & 0 \\ 20.1 & 20.1 & 53.2 & 0 & 0 & 0 \\ 0 & 0 & 0 & 22.6 & 0 & -9.7 \\ 9.7 & -9.7 & 0 & 0 & 22.6 & 0 \\ 0 & 0 & 0 & -9.7 & 0 & 33.1 \end{bmatrix}$$

Polar optical phonon frequency (THz) = 7.15

Competing Phases Analysis

Table S2 Calculated formation energies of stable competing phases for $Sc_2Si_2Te_6$ with HSE06+SOC level.

System	Space group	Formation energy (eV/atom)
Si	$Fd\bar{3}m$	0
Te	$P3_121$	0
Sc_2Te_3	$P\bar{1}$	-7.16
Sc_5Si_3	$P6_3/mcm$	-6.71
Sc_8Te_3	C2/m	-8.61
ScSi	Cmcm	-1.74
ScTe	$P6_3/mmc$	-2.58
$Sc_2Si_2Te_6$	$R\bar{3}m$	-7.98

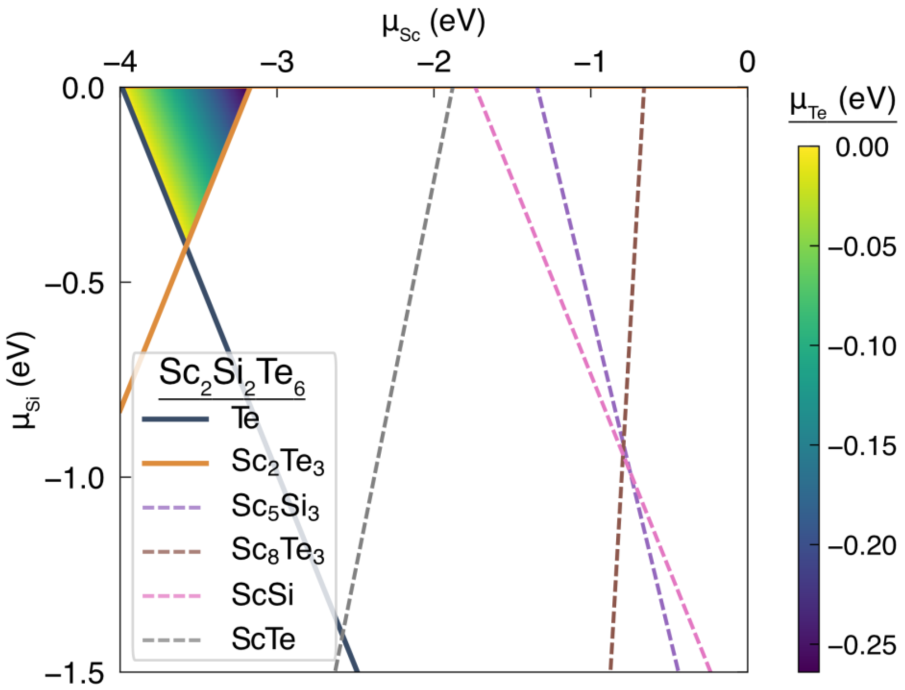

Figure S1 The calculated chemical stability regions (chemical potentials) for $Sc_2Si_2Te_6$, using HSE06+SOC, plotted using doped.[1] The element Te is set as the dependent variable, with its chemical potential indicated by the colourmap. Bordering lines correspond to the competing phases within the $Sc_2Si_2Te_6$ chemical space.

To assess the thermodynamic stability of $Sc_2Si_2Te_6$, the formation energies of all stable competing phases in the Sc-Si-Te phase diagram were computed using HSE06+SOC, as tabulated in Table S2. Figure S1 plots the computed chemical stability region of $Sc_2Si_2Te_6$, which delineates the competing phases and associated chemical potentials within the Sc-Si-Te phase space. The analysis indicates that $Sc_2Si_2Te_6$ is thermodynamically stable over a relatively narrow μ_{Te} range, a moderate μ_{Si} range, and an extended μ_{Sc} range with the chemical potential limits set by elemental Te, Si and Sc_2Te_3 as the bordering competing phases. The size of the chemical stability region provides an indication of the synthesizability and expected secondary phases for a given compound. The relatively large chemical stability region exhibited by $Sc_2Si_2Te_6$ suggests ready synthesizability for this material, as witnessed experimentally.[2]

Additional Electronic Analysis

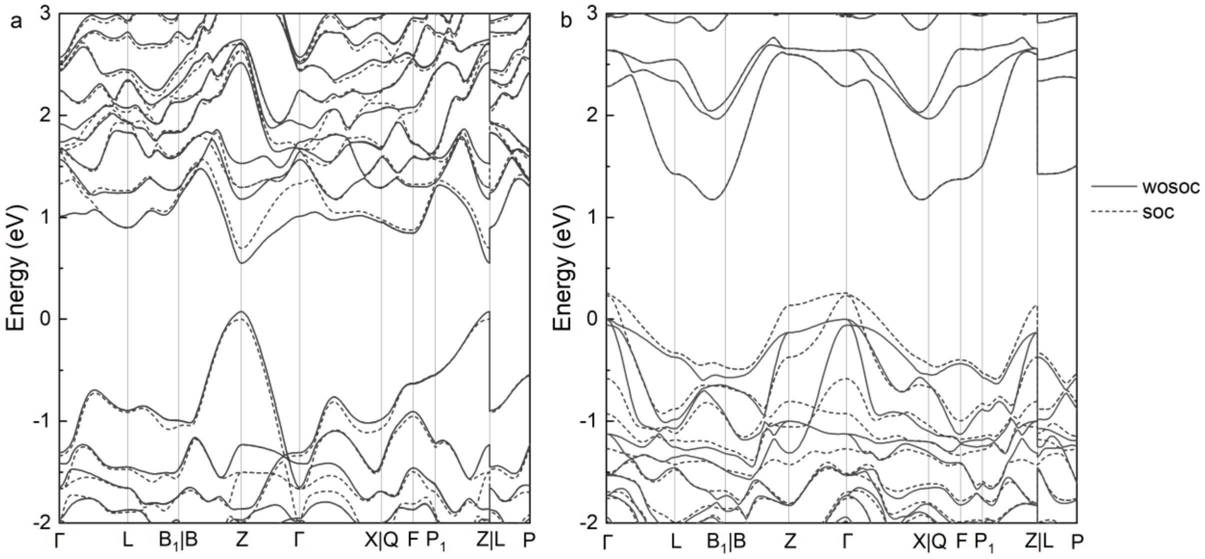

Figure S2 Calculated HSE06 band structures of (a) $Sb_2Si_2Te_6$ and (b) $Sc_2Si_2Te_6$ with and without SOC effects, shown in solid and dashed lines, respectively. The VBM without SOC is set to zero.

Given the presence of heavy elements such as Sb and Te, the band structures with and without SOC are compared in Figure S2. The results demonstrate that the SOC-induced band splitting is particularly pronounced in the VBM of $Sc_2Si_2Te_6$ due to the Te-*p* orbital splitting, as expected. As shown in Figure S2, SOC splits the threefold-degenerate Te-*p* VBM in $Sc_2Si_2Te_6$ into a double-degenerate VBM and a single split-off band ~0.9 eV below the VBM – as also witnessed in CdTe.[3] Without SOC, the band gap is 0.7 and 1.17 eV for $Sb_2Si_2Te_6$ and $Sc_2Si_2Te_6$, respectively, indicating the significant role of relativistic effects in the electronic structure of these compounds.

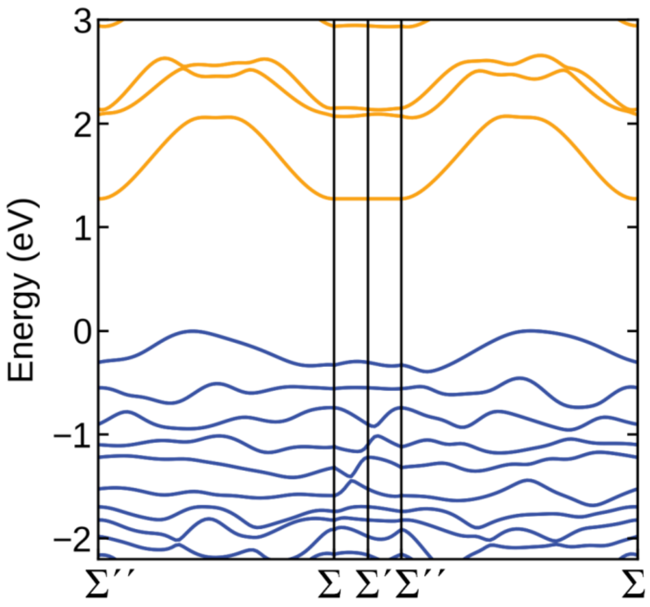

Figure S3 Calculated electronic band structure of $Sc_2Si_2Te_6$ using HSE06+SOC, along the linear path between *k*-points Σ, Σ′ and Σ′′ at $(^1/_2, -^1/_6, ^1/_6)$, $(^1/_3, -^1/_3, 0)$ and $(^1/_6, -^1/_2, -^1/_6)$ in fractional reciprocal coordinates (see main text). The VBM is set to 0 eV. Note that the conduction band from Σ to Σ′′ is too flat for the analytical effective mass approximation to be applicable.

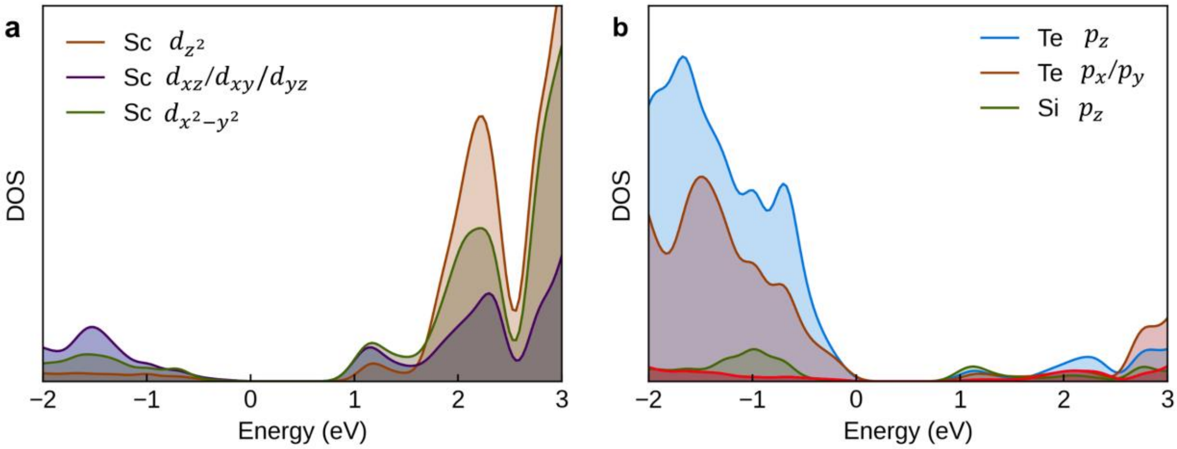

Figure S4 *lm*-decomposed electronic DOS of (a) Sc-*d* orbitals and (b) Te/Si-*p* orbitals in $Sc_2Si_2Te_6$, calculated using HSE06+SOC. VBM is set to 0 eV.

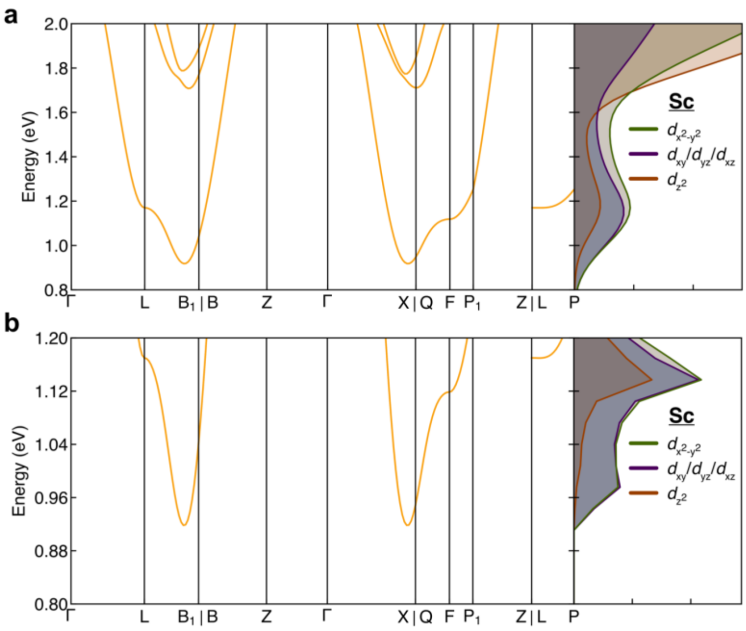

Figure S5 Calculated electronic band structure and *lm*-decomposed orbital-projected DOS of $Sc_2Si_2Te_6$ using HSE06+SOC, in the region of the conduction band minimum (CBM). The VBM is set to 0 eV. (a) shows a wider energy range with some small Gaussian broadening of the DOS, while a narrower energy range and no Gaussian broadening is shown in (b) for clarity.

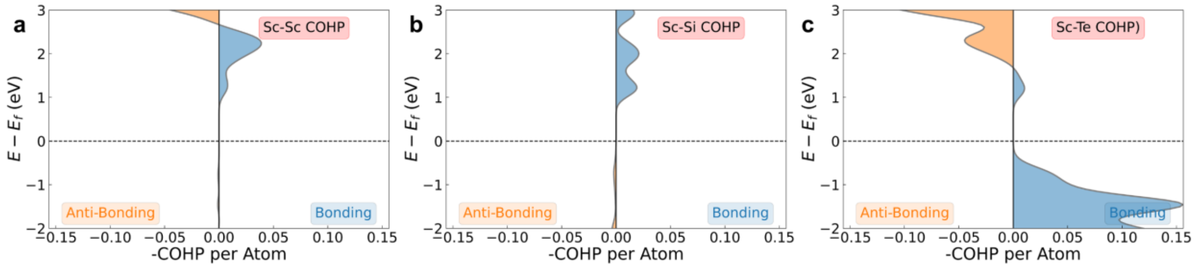

Figure S6 Crystal Orbital Hamilton Population (COHP) as a function of energy for orbital interactions between (a) Sc-Sc, (b) Sc-Si and (c) Sc-Te in $Sc_2Si_2Te_6$, calculated using HSE06+SOC. Negative COHP values (blue) indicate energy-lowering, bonding-type interactions, while positive values (orange) indicate anti-bonding character.

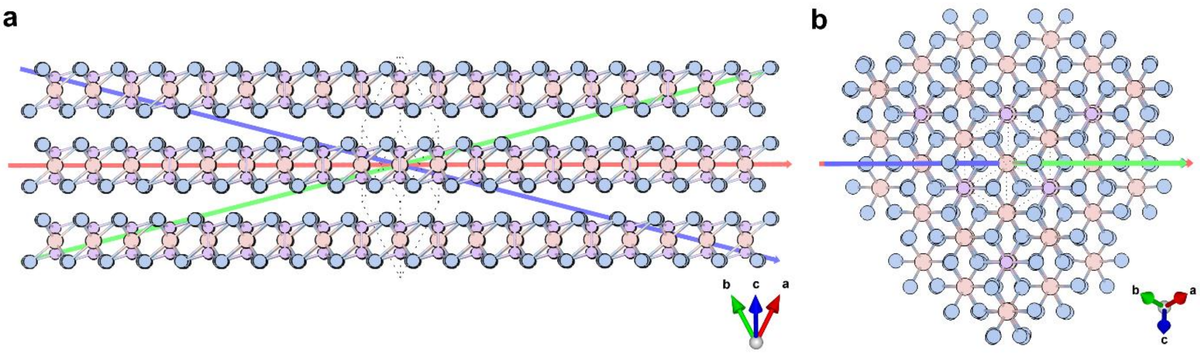

Figure S7 Real space directions corresponding to the Σ (green), Σ′ (red) and Σ′′ (blue) *k*-points in $Sc_2Si_2Te_6$ (i.e. the location of the 6-fold degenerate conduction band valleys), looking along the layers (a) and perpendicular to the layers (b). Filled black circles indicate full periods (i.e. in-phase positions) in the *k*-point phase factor and white circles indicate half periods (i.e. anti-phase positions), taking the central Sc atom as the origin.

Scattering rates analysis

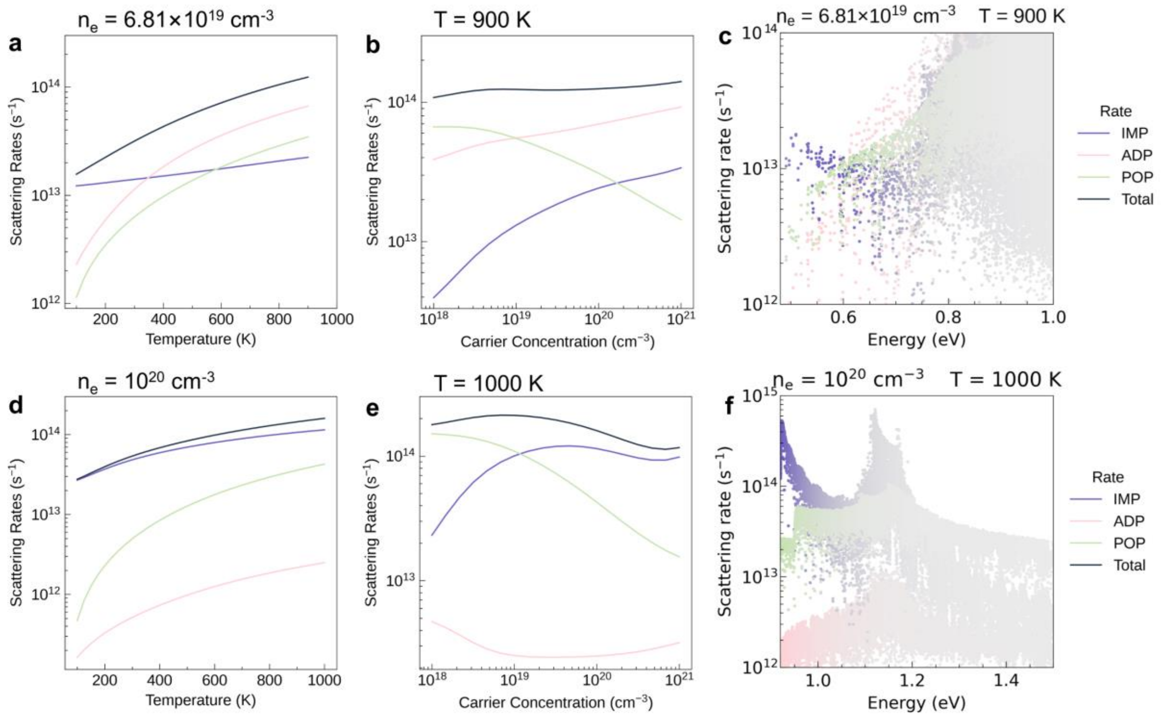

Figure S8 Average scattering rates for n-type (top panel) $Sb_2Si_2Te_6$ and (bottom panel) $Sc_2Si_2Te_6$ with carrier concentrations / temperatures fixed to the values giving the maximum predicted *ZT*. The scattering rates as a function of (a, d) temperature, (b, e) carrier concentration, and (c, f) energy relative to the CBM. $Sb_2Si_2Te_6$ and $Sc_2Si_2Te_6$ have demonstrated thermal stability up to 920 K and 1023 K,[2, 4] respectively, and so these ranges are used in our analysis. In (c, f), the colour strengths reflect the availability of carrier scattering channels through the band occupancies (and thus the weighted impacts of the scattering rates on the overall mobilities), as given by the derivative of the Fermi-Dirac distribution function.

Figures S8a and S8d display the average electron scattering rates as a function of temperature with fixed carrier concentration for n-type $Sb_2Si_2Te_6$ and $Sc_2Si_2Te_6$. The corresponding scattering rates for p-type systems are presented in Figure S9. Lower scattering rates yield higher electrical conductivity. As depicted in Figures S8a and S8d, the total scattering rate increases with temperature due to increased carrier-phonon interactions (polar optical phonon (POP) and acoustic deformation potential (ADP) scattering) at higher temperatures. Notably, $Sb_2Si_2Te_6$ exhibits a lower total scattering rate compared to $Sc_2Si_2Te_6$, primarily due to the larger high-frequency dielectric constants (due to the smaller band gap), which is beneficial for achieving higher electrical conductivity. On the other hand, Figures S8b and S8e illustrate the scattering rates as a function of carrier concentration with fixed temperature. At low carrier concentrations, POP scattering governs the scattering rates for both $Sb_2Si_2Te_6$ and $Sc_2Si_2Te_6$. POP scattering arises from the vibration of optical phonon modes, leading to the formation of dipoles, which scatter charge carriers. As carrier

concentration rises, ADP becomes the predominant scattering mechanism for n-type $Sb_2Si_2Te_6$, while ionised impurity (IMP) scattering starts to dominate in n-type $Sc_2Si_2Te_6$. This difference can be attributed to the relatively large deformation potential in $Sb_2Si_2Te_6$ (5.74 eV in the *xy* plane and 2.42 eV along the *z*-direction, compared to 1.56 eV and 0.91 eV for *xy* and *z* directions in $Sc_2Si_2Te_6$). The ADP scattering mechanism arises from the interaction between electrons and acoustic phonons. When lattice vibrations occur, they cause a local strain in the crystal structure, which perturbs the energy bands and scatters the charge carriers. On the other hand, IMP scattering arises from the presence of charged defects (dopants) which act as scattering centres within the lattice. The charged impurities induce the free carrier population in doped semiconductors while also contributing to carrier scattering, and so IMP scattering increases with doping concentration until it reaches a plateau. IMP scattering is relatively independent of temperature. A similar trend is observed for p-type $Sb_2Si_2Te_6$ and $Sc_2Si_2Te_6$, as illustrated in Figures S9b and S9e. At low carrier concentrations, POP is the primary scattering mechanism, while as carrier concentration increases, IMP and ADP scattering mechanisms become more significant. Figures S8c and S8f present the relationship between scattering rates and energy. The colour strengths reflect the availability of carrier scattering channels through the band occupancies (and thus the weighted impacts of the scattering rates on the overall mobilities), as given by the derivative of the Fermi-Dirac distribution function. In both compounds under conditions which maximise *ZT*, IMP scattering exhibits the highest scattering rates for states near the band edge, while POP and ADP exhibit faster scattering rates for higher energy states.

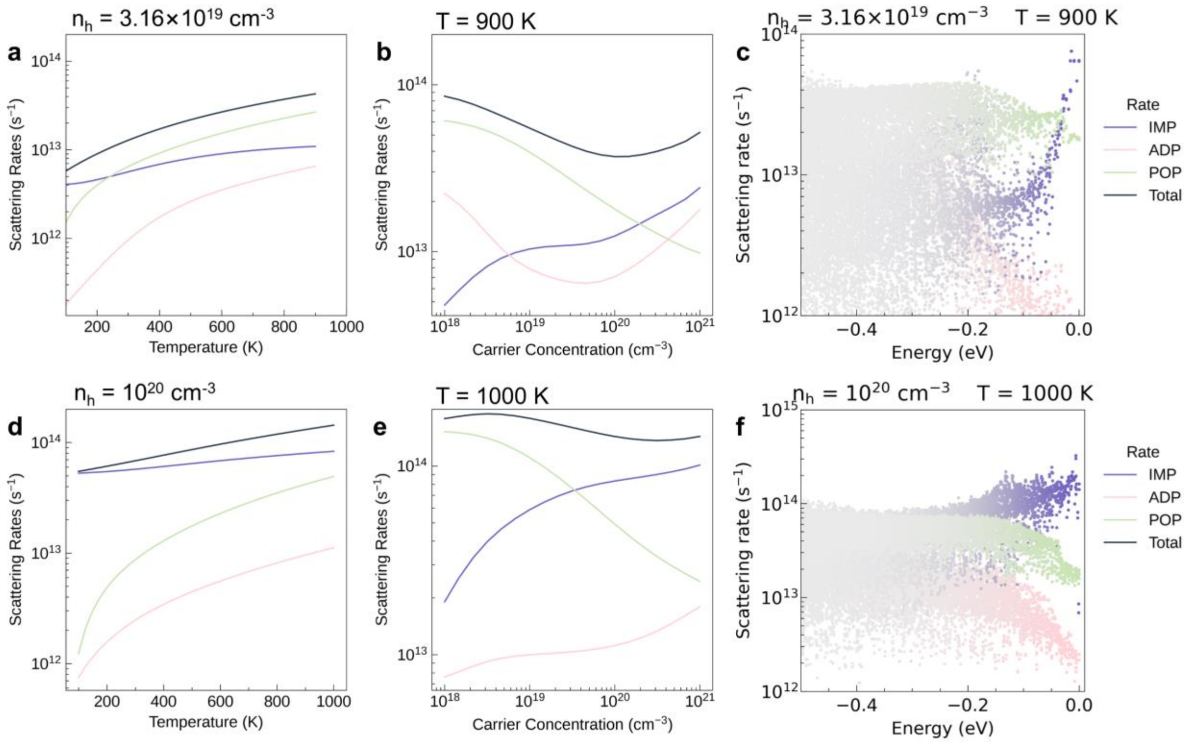

Figure S9 Average scattering rates for p-type (top panel) $Sb_2Si_2Te_6$ and (bottom panel) $Sc_2Si_2Te_6$ with carrier concentrations / temperatures fixed to the values giving the maximum predicted *ZT*. The scattering rates as a function of (a, d) temperature, (b, e) carrier concentration, and (c, f) energy relative to the VBM. $Sb_2Si_2Te_6$ and $Sc_2Si_2Te_6$ have demonstrated thermal stability up to 920 K and 1023 K,[2, 4] respectively, and so these ranges are used in our analysis. In (c, f), the colour strengths reflect the availability of carrier scattering channels through the band occupancies (and thus the weighted impacts of the scattering rates on the overall mobilities), as given by the derivative of the Fermi-Dirac distribution function.

Additional Electronic Transport Properties Analysis

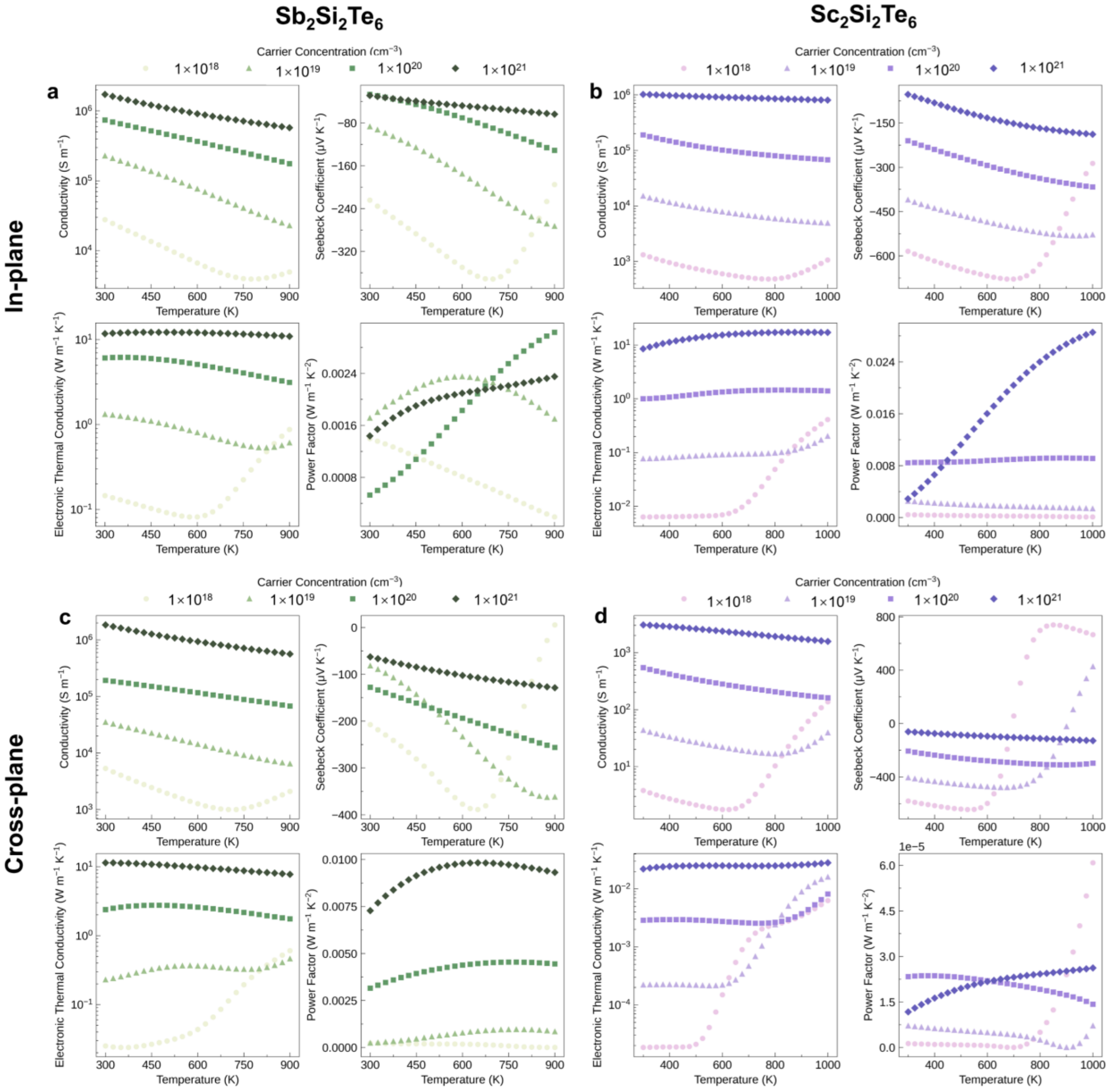

Figure S10 Calculated electronic transport properties as a function of temperature for n-type $Sb_2Si_2Te_6$ (a, c) and $Sc_2Si_2Te_6$ (b, d) along both the in-plane (top panel) and cross-plane (bottom panel) directions with four different carrier concentrations.

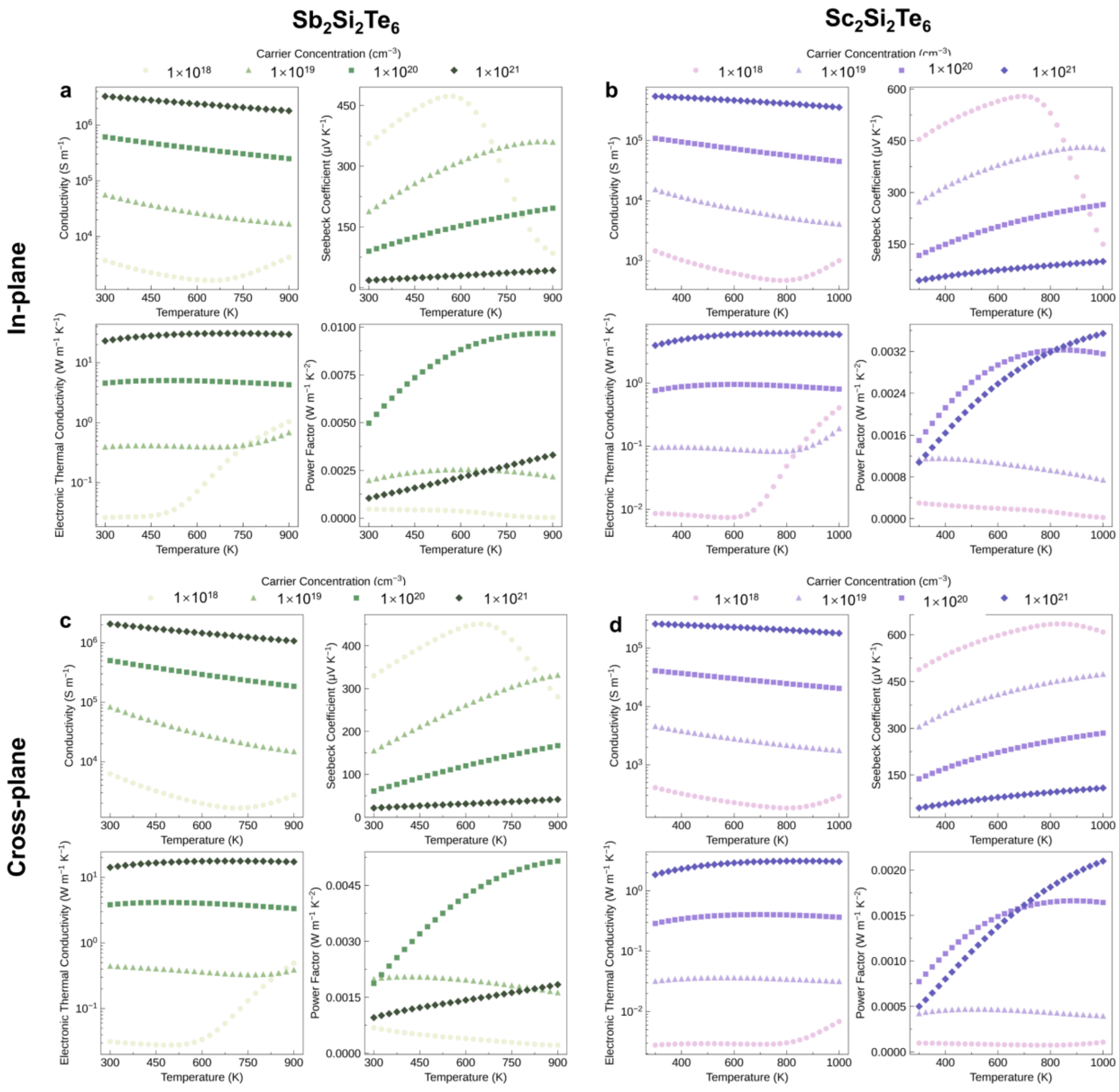

Figure S11 Calculated electronic transport properties as a function of temperature for p-type $Sb_2Si_2Te_6$ (a, c) and $Sc_2Si_2Te_6$ (b, d) along both the in-plane (top panel) and cross-plane (bottom panel) directions with four different carrier concentrations.

The abnormal behaviour of the Seebeck coefficient observed in both n-type and p-type $Sb_2Si_2Te_6$ ($Sc_2Si_2Te_6$) at a low carrier concentration of 1×10^{18} cm^{-3} and elevated temperatures, can be ascribed to the ambipolar conduction effect. The distinctive signature of the ambipolar conduction effect is the increased electrical conductivity and electronic thermal conductivity as well as the decreased absolute value of the Seebeck coefficient, observed in narrow-gap semiconductors at high temperatures and low carrier concentrations.[5, 6] The reversal in the sign of the Seebeck coefficient at high temperatures indicates the excitation of minority carriers and the ensuing competition between majority and minority charge carriers. The presence of two distinct charge carriers results in deviation of the ideal $S \propto n^{-\frac{2}{3}}$ relationship,

yielding a reduced Seebeck coefficient at low carrier concentration. As the carrier concentration increases, the Seebeck coefficient is again determined by the majority of carriers, conforming to the $S \propto n^{-\frac{2}{3}}$ relationship. Analogous phenomena have been reported in other materials as well.[5, 7]

Phonon dispersion and phonon DOS

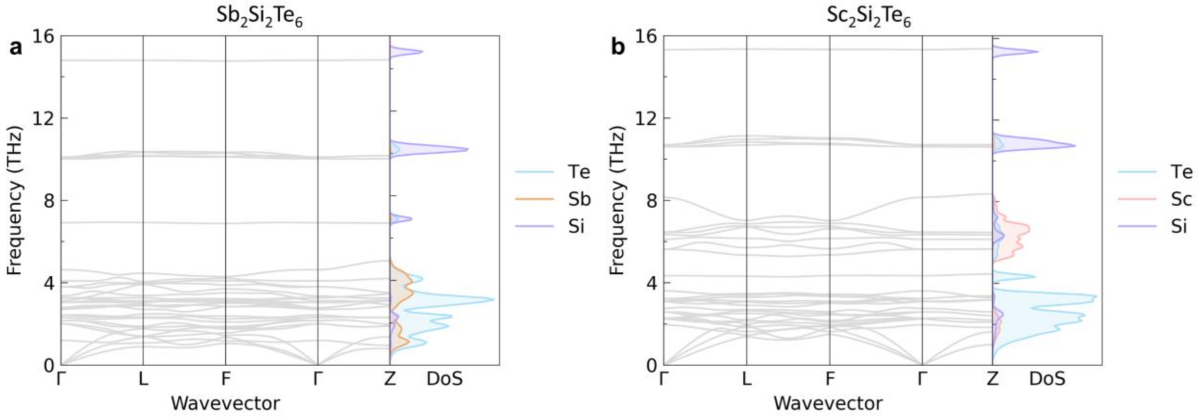

Figure S12 Phonon dispersion and phonon DOS for (a) $Sb_2Si_2Te_6$ and (b) $Sc_2Si_2Te_6$. The *k*-point path uses the Bradley-Cracknell formalism.[8]

The phonon dispersion presented in Figure S12 demonstrates the dynamical stability of $Sb_2Si_2Te_6$ and $Sc_2Si_2Te_6$ without any imaginary frequencies. Flat phonon bands are indicative of small phonon group velocities and are thus favourable for low lattice thermal conductivity.[9] The phonon dispersion in both $Sb_2Si_2Te_6$ and $Sc_2Si_2Te_6$ shows mostly flat modes, which tend to generate low lattice thermal conductivity. In both systems, the greatest contribution of acoustic modes and low-frequency optical modes (below 4 THz) originate from the heavy Te atoms, as indicated by the PDOS. In $Sb_2Si_2Te_6$, the high-frequency optical modes are mainly dominated by Si atoms. Instead, in the case of $Sc_2Si_2Te_6$, the optical modes range from 4 to 8 THz, primarily originating from Sc atoms, with higher frequency optical modes governed by the Si atoms. The considerable mass of Sb (121.76 a.u.), which is comparable to Te (127.60 a.u.) and much heavier than Sc (44.96 a.u.), leads to a significant downshift in Sb phonon mode frequencies into the 0-5 THz range. This results in a strong coupling (and thus phonon scattering) between the acoustic and low-frequency optical modes.

Additional Thermal Transport Properties Analysis

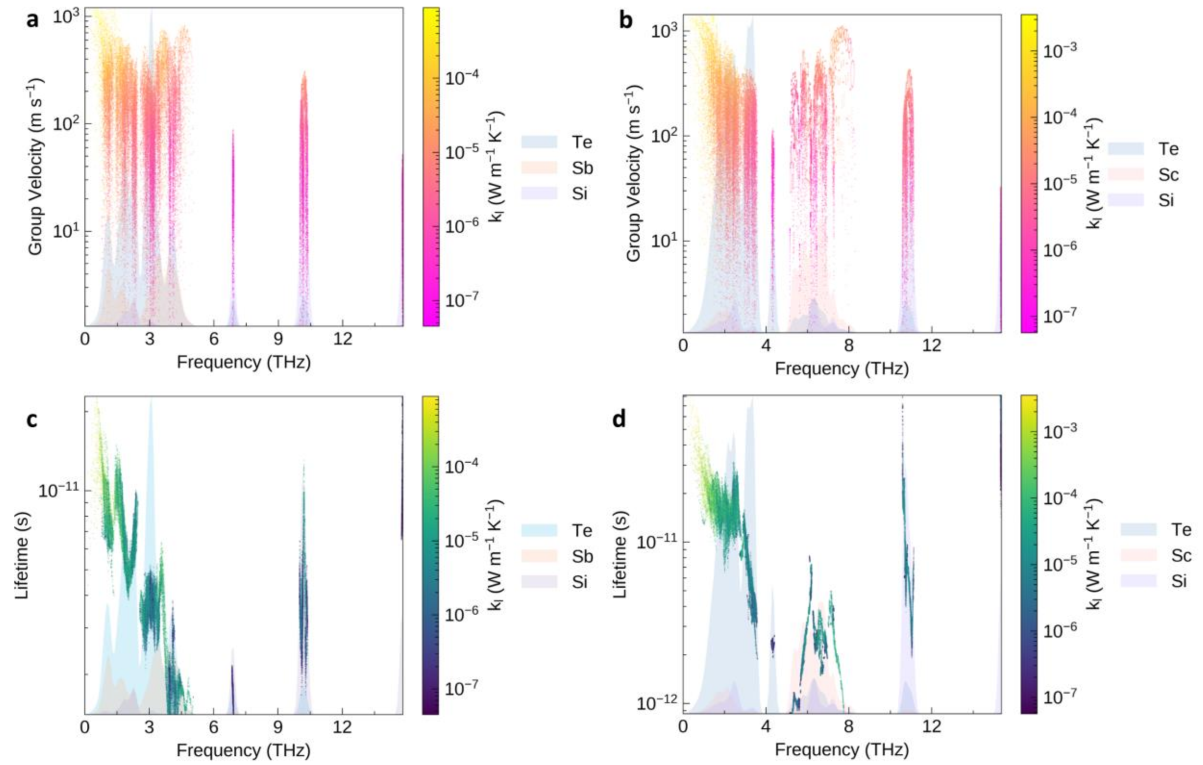

Figure S13 Analysis of the isotropically-averaged modal group velocity ν_λ and lifetime τ_λ for $Sb_2Si_2Te_6$ (a, c) and $Sc_2Si_2Te_6$ (b, d) at 300 K. The data points are colour-coded by the modal contributions to lattice thermal conductivity κ_l, from pink to yellow for $Sb_2Si_2Te_6$ and purple to yellow for $Sc_2Si_2Te_6$. The faded background is the phonon DOS.

To gain deeper insights into the thermal transport mechanism, we analysed the phonon group velocity and the phonon lifetime as functions of frequency at 300 K (Figure S13). Within the single-mode relaxation time approximation, the lattice thermal conductivity κ_l is calculated as the sum of contributions from individual phonon modes (λ) using the formula, $\kappa_l = \frac{1}{NV}\sum_\lambda C_\lambda \, \nu_\lambda \otimes \nu_\lambda \tau_\lambda$, where N represents the number of wavevectors in the summation (equivalent to the number of unit cells in the crystal), V is the unit cell volume, C_λ denotes the modal heat capacities, ν_λ represents the modal group velocities and τ_λ stands for the phonon lifetimes. The contribution of individual modes to κ_l can be examined from scatter plots of the three modal quantities in the equation against the phonon frequency.

Most phonon group velocities in $Sb_2Si_2Te_6$ and $Sc_2Si_2Te_6$ span a range of ~1 to ~10^3 m s^{-1}, as illustrated in Figures S13a and S13b. In $Sb_2Si_2Te_6$, the highest group velocities are observed between 1-5 THz, while in $Sc_2Si_2Te_6$, high group velocities are found for a broader range from 1-3 and 5-8 THz. The relatively lower proportion of phonon modes with high group velocities in $Sb_2Si_2Te_6$ can be ascribed to the localization of phonon modes resulting

from the presence of Sb. On the other hand, phonon lifetimes are inversely related to the phonon linewidths Γ_λ *via* $\tau_\lambda = \frac{1}{2\Gamma_\lambda}$, where Γ_λ values are determined by energy- and momentum-conserving three-phonon scattering processes. Unlike the phonon group velocities, the phonon lifetimes are scalar quantities. In $Sb_2Si_2Te_6$, approximately 85% of the modes have lifetimes less than 10 ps, while in $Sc_2Si_2Te_6$, about 62% of the modes exhibit lifetimes shorter than 10 ps. The relatively shorter phonon lifetimes in $Sb_2Si_2Te_6$ indicate enhanced phonon-phonon scattering rates and subsequent lower lattice thermal conductivity. Analyses in Figure S13 indicate that $Sb_2Si_2Te_6$ and $Sc_2Si_2Te_6$ exhibit a comparable range of group velocities, while the shorter phonon lifetimes in $Sb_2Si_2Te_6$ support its lower lattice thermal conductivity.

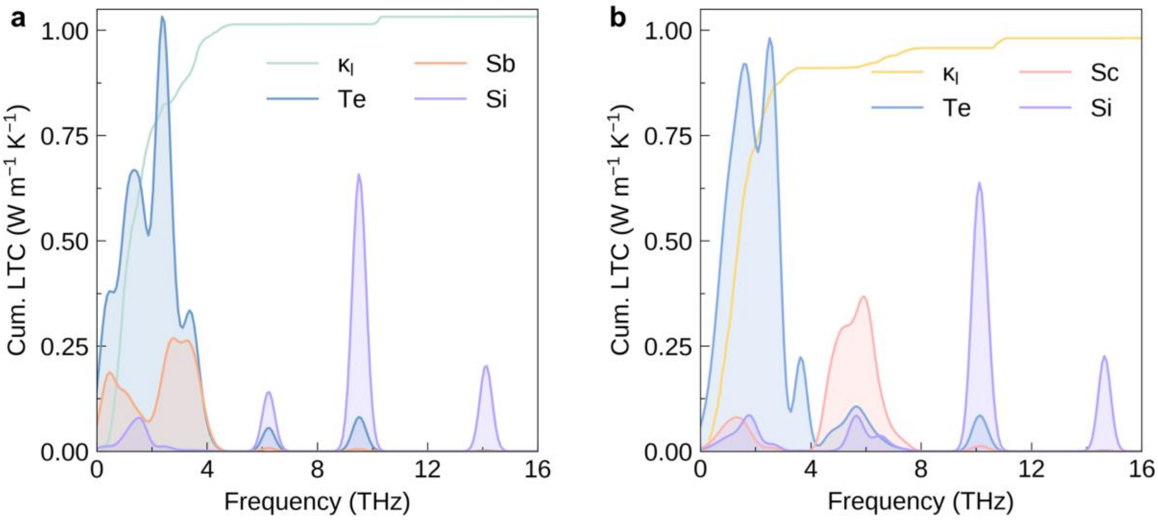

Figure S14 Cumulative lattice thermal conductivity of (a) $Sb_2Si_2Te_6$ and (b) $Sc_2Si_2Te_6$ at 300 K as a function of frequency against the phonon density of states.

Examining the cumulative lattice thermal conductivity as a function of phonon frequency illustrates the contribution of different phonon modes to the total lattice thermal conductivity (Figure S14). More than 90% of lattice thermal conductivity comes from the low-frequency phonon modes in both systems. To be specific, at 300 K, the contributions of acoustic phonon modes to lattice thermal conductivity are ~58% and ~71% for $Sb_2Si_2Te_6$ (< 1.5 THz) and $Sc_2Si_2Te_6$ (< 1.8 THz), respectively. The low-frequency optical phonon modes contribute ~32% and ~19% to lattice thermal conductivity for $Sb_2Si_2Te_6$ (1.5 − 5 THz) and $Sc_2Si_2Te_6$ (1.8 − 5 THz), respectively. Furthermore, the thermal conductivity contributions peak is observed earlier in the $Sc_2Si_2Te_6$, as the low-frequency phonon modes in $Sc_2Si_2Te_6$ are supported mainly by Te rather than Te and Sb in $Sb_2Si_2Te_6$, which reduces the phonon-phonon scattering, elongating the phonon lifetimes.

Predicted Thermoelectric figure of merit *ZT*

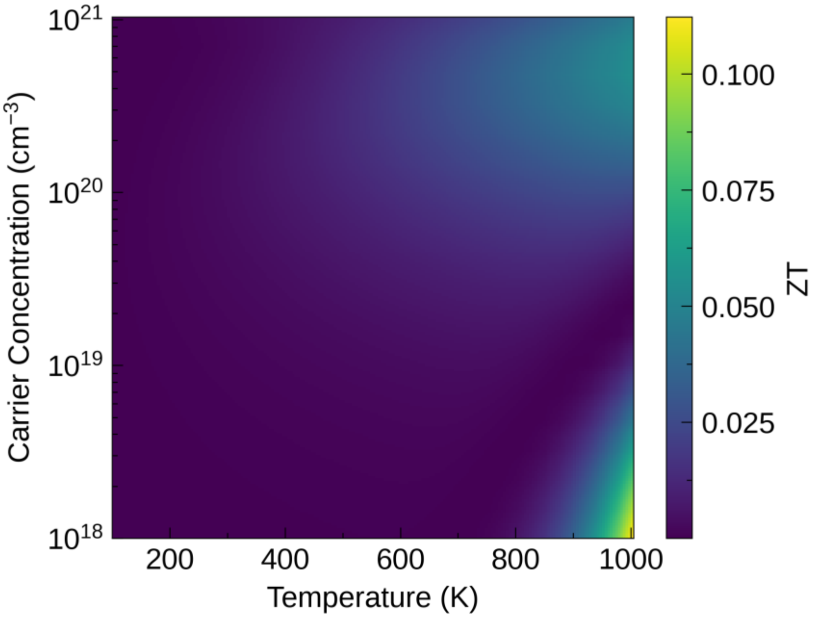

Figure S15 Predicted thermoelectric figure of merit *ZT* of n-type $Sc_2Si_2Te_6$ against temperature and carrier concentration along the cross-plane direction. The lightest lobe in the lower right corner represents $Sc_2Si_2Te_6$ has become p-type system due to the ambipolar conduction effect.

Convergence of the Electronic Transport Properties with Respect to the Interpolation Factor

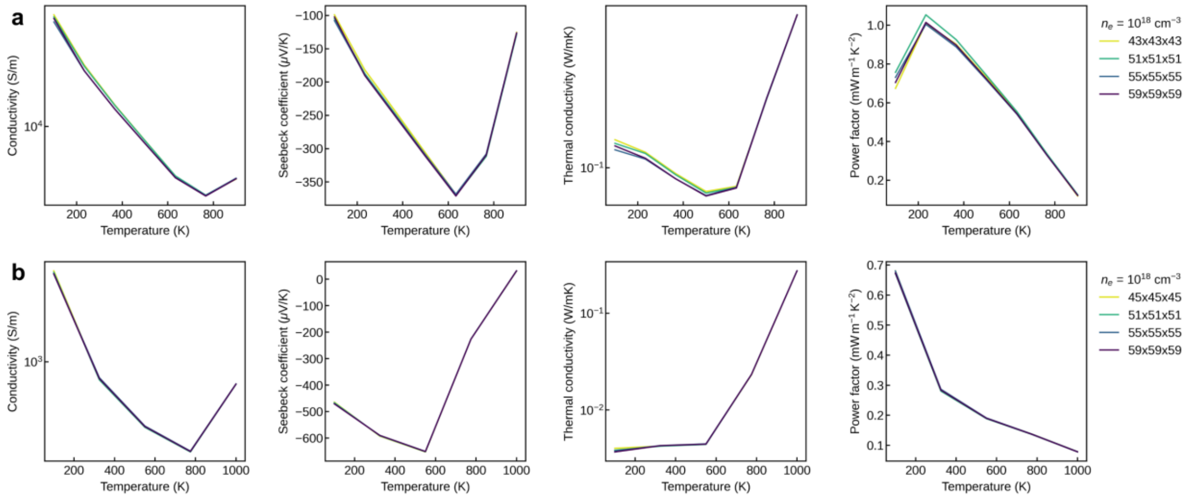

Figure S16 Electronic transport properties for (a) $Sb_2Si_2Te_6$ and (b) $Sc_2Si_2Te_6$ as a function of temperature, calculated over different Fourier interpolated mesh densities.

Phonon supercell convergence testing

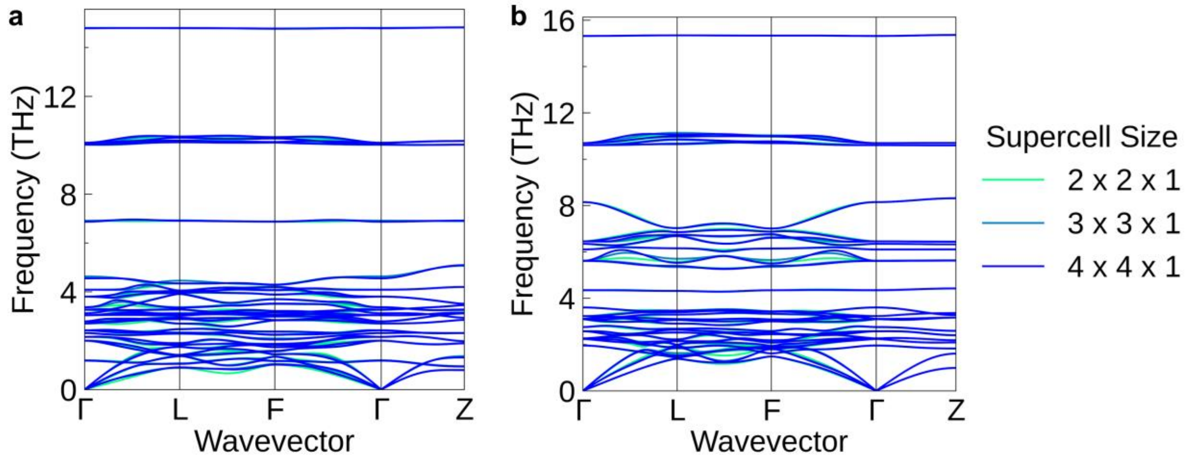

Figure S17 Convergence of phonon dispersion for (a) $Sb_2Si_2Te_6$ and (b) $Sc_2Si_2Te_6$ with different supercell sizes. The critical aspects of the phonon dispersion are converged with the 2×2×1 supercell. The *k*-point path uses the Bradley-Cracknell formalism.[8]

Convergence of κ_l with q-point Sampling Mesh

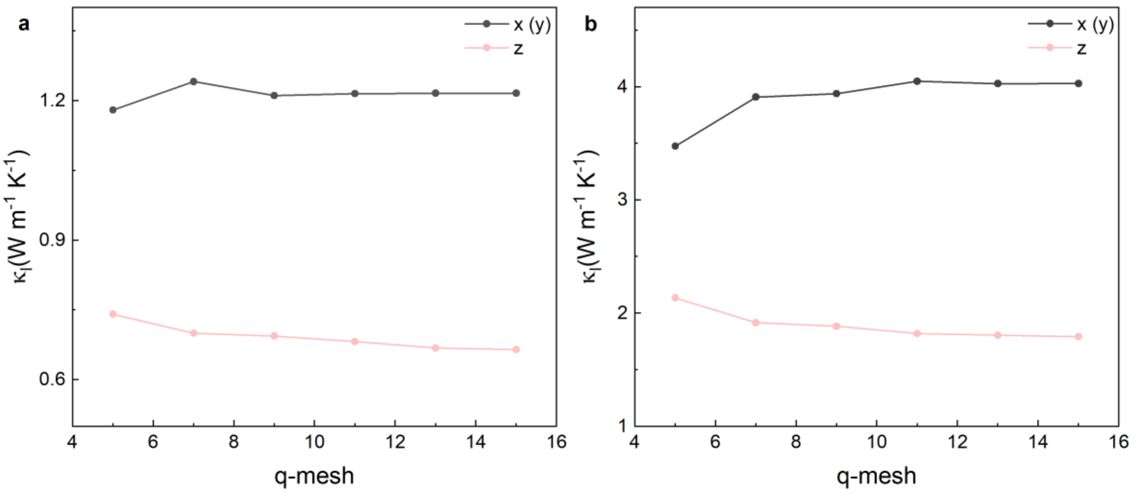

Figure S18 Convergence of lattice thermal conductivity κ_l at 300 K with respect to the q-point mesh sampling density for (a) $Sb_2Si_2Te_6$ and (b) $Sc_2Si_2Te_6$.

Band Alignment Calculations

The band alignment was calculated according to the core-vacuum alignment scheme derived by Wei and Zunger.[10]

The ionisation potential (IP) and electron affinity (EA) were calculated as:

$$IP = (E_{vac} - E_{core,\ slab}) - (E_{VBM} - E_{core,\ bulk}) \quad (1)$$

$$EA = IP - E_g \quad (2)$$

where E_{vac} and $E_{core, slab}$ are the energies of the vacuum and the Te 1*s* core level in the bulk-like surface slab, respectively, E_{VBM} is the valence band maximum of the bulk and $E_{core, bulk}$ is the bulk Te 1*s* core energy.

To calculate the energies of the surface slab, surfaxe package[11] was used to cleave the (001) slab from the HSE06-relaxed conventional unit cell.

Reference

(1) Kavanagh, S. R.; Squires, A. G.; Nicolson, A.; Mosquera-Lois, I.; Ganose, A. M.; Zhu, B.; Brlec, K.; Walsh, A.; Scanlon, D. O. doped: Python toolkit for robust and repeatable charged defect supercell calculations. *J. Open Source Softw.* **2024**, *9*, 6433.
(2) Pielnhofer, F.; Bette, S.; Eger, R.; Duppel, V.; Nuss, J.; Dolle, C.; Dinnebier, R. E.; Lotsch, B. V. The Stacking Faulted Nature of the Narrow Gap Semiconductor $Sc_2Si_2Te_6$. *Z. Anorg. Allg. Chem.* **2022**, *648*, e202200234.
(3) Kavanagh, S. R.; Walsh, A.; Scanlon, D. O. Rapid Recombination by Cadmium Vacancies in CdTe. *ACS Energy Lett.* **2021**, *6*, 1392-1398.
(4) Luo, Y.; Cai, S.; Hao, S.; Pielnhofer, F.; Hadar, I.; Luo, Z.-Z.; Xu, J.; Wolverton, C.; Dravid, V. P.; Pfitzner, A.; et al. High-Performance Thermoelectrics from Cellular Nanostructured $Sb_2Si_2Te_6$. *Joule* **2020**, *4*, 159-175.
(5) Shahi, P.; Singh, D. J.; Sun, J. P.; Zhao, L. X.; Chen, G. F.; Lv, Y. Y.; Li, J.; Yan, J. Q.; Mandrus, D. G.; Cheng, J. G. Bipolar Conduction as the Possible Origin of the Electronic Transition in Pentatellurides: Metallic vs Semiconducting Behavior. *Phys. Rev. X* **2018**, *8*, 021055.
(6) Chen, Z.; Zhang, X.; Ren, J.; Zeng, Z.; Chen, Y.; He, J.; Chen, L.; Pei, Y. Leveraging bipolar effect to enhance transverse thermoelectricity in semimetal Mg_2Pb for cryogenic heat pumping. *Nat. Commun.* **2021**, *12*, 3837.
(7) Zhao, L. D.; Lo, S. H.; Zhang, Y.; Sun, H.; Tan, G.; Uher, C.; Wolverton, C.; Dravid, V. P.; Kanatzidis, M. G. Ultralow thermal conductivity and high thermoelectric figure of merit in SnSe crystals. *Nature* **2014**, *508*, 373-377.
(8) Bradley, C.; Cracknell, A. *The mathematical theory of symmetry in solids: representation theory for point groups and space groups*; Oxford University Press, 2010.
(9) Einhorn, M.; Williamson, B. A. D.; Scanlon, D. O. Computational prediction of the thermoelectric performance of LaZnOPn (Pn = P, As). *J. Mater. Chem. A* **2020**, *8*, 7914-7924.
(10) Wei, S.-H.; Zunger, A. Calculated natural band offsets of all II–VI and III–V semiconductors: Chemical trends and the role of cation d orbitals. *Appl. Phys. Lett.* **1998**, *72*, 2011-2013.
(11) Brlec, K.; Davies, D. W.; Scanlon, D. O. Surfaxe: Systematic surface calculations. *J. Open Source Softw.* **2021**, *6*, 3171.
